# High Dose Vitamin D3 Supplementation Is Not Associated With Lower Mortality in Critically Ill Patients: A Meta-Analysis of Randomized Control Trials

**DOI:** 10.3389/fnut.2022.762316

**Published:** 2022-05-04

**Authors:** Zhiwei Gao, Jianfeng Xie, Cong Li, Ling Liu, Yi Yang

**Affiliations:** ^1^Jiangsu Provincial Key Laboratory of Critical Care Medicine, Department of Critical Care Medicine, School of Medicine Southeast University, Zhongda Hospital, Nanjing, China; ^2^Department of Emergency Intensive Care Unit, The Affiliated Huaian No. 1 People's Hospital of Nanjing Medical University, Huai'an, China

**Keywords:** vitamin D3, cholecalciferol, intensive care unit (ICU), parenteral nutrition, prognosis

## Abstract

**Background:**

Vitamin D deficiency is a common condition in critically ill patients. A high dose of vitamin D3 can rapidly restore vitamin D levels. The aim of this meta-analysis was to synthesize the results from up-to-date randomized control trials (RCT) and validate the effect of vitamin D3 in critically ill patients.

**Study Methods:**

Several databases, including PubMed, Web of Science, EMBASE, and the Cochrane Central database, were searched up to December 4th, 2020. All RCTs that investigated the use of a high dose of vitamin D3 in critically ill patients and reported mortality data were included in the meta-analysis. The primary outcome was the mortality truncated to day 28 and day 90.

**Results:**

A total of 10 RCTs enrolling 2058 patients were finally included. The use of a high dose of vitamin D3 in critically ill patients could not decrease the mortality truncated to day 28 (RR 0.93, 95% CI 0.78–1.11, *P* = 0.43) or day 90 (RR 0.91, 95% CI 0.79–1.05, *P* = 0.21). A high dose of vitamin D3 could significantly reduce the ventilator days (MD −9.38, 95%CI −13.44 to −5.31, *P* < 0.001), but there were no statistic difference in length of ICU stay (MD −2.76, 95% CI −6.27 to 0.74, *P* = 0.12) and hospital stay (MD −2.42, 95% CI −6.21 to 1.36, *P* = 0.21). No significant difference was observed in adverse events between the vitamin D3 group and the placebo group.

**Conclusion:**

The use of high dose vitamin D3 was not associated with decreased mortality in critically ill patients, but could significantly reduce the ventilator days.

**Systematic Review Registration:**

https://www.crd.york.ac.uk/prospero/, identifier: CRD42020179195.

## Introduction

Vitamin D deficiency is a common condition in critically ill admissions, with a prevalence between 79 and 89% (1, 2). Evidence from conventional studies shows that vitamin D deficiency in critically ill patients is associated with a higher incidence of sepsis development (2), Sequential Organ Failure Assessment (SOFA) score, and a longer duration of ICU stay and mechanical ventilation (3). A recent cross-sectional study at the clinical ICU of University Hospital also verified that low serum 25-hydroxyvitamin D (25[OH]D) concentrations were significantly associated with the Charlson Comorbidity Index, which is a prognostic indicator, and clinical complications (4).

Vitamin D3 is the most extensively used type of vitamin D in clinical situations. A series of trials confirmed that an ultrahigh loading dose of vitamin D3 (single bolus dose from 400,000 to 540,000 IU) could rapidly restore vitamin D levels, and very limited side effects were reported (5–8). High-dose vitamin D3 for rapidly restoring vitamin D levels has been shown to be beneficial to critically ill patients. A randomized double-blind placebo clinical trial confirmed that a single bolus dose of 300,000 IU vitamin D3 for patients with ventilator-associated pneumonia helped to reduce the serum PCT concentrations on day 7 (9). Another multicenter RCT indicated that a single high dose of cholecalciferol significantly decreased the postoperative pulmonary vascular permeability index and could prevent lung injury in patients undergoing esophagectomy (10). Additionally, vitamin D may have beneficial effects on the immune response to acute inflammation and hospital infection, cardiogenic function and other critically ill conditions (11, 12).

The effect of high-dose vitamin D3 application on critically ill patient mortality is inconsistent. The VITdAL-ICU RCT showed administration of high dose vitamin D3 (single enteral dose of vitamin D3 540,000 IU and monthly maintenance dose of 90,000 IU for 5 months) did not reduce hospital or 6-month mortality (8). But a post-hoc analysis from the VITDAL-ICU study excluding patients who died early revealed that high dose of vitamin D was associated with reduction in 28 day mortality (13). And then previous meta-analysis found that vitamin D3 supplementation might be associated with a reduction in mortality in critically ill patients (32% vs. 40%, 0.7 [95% CI, 0.50–0.98], *P* = 0.04) (14). However, the VIOLET trial showed that early high dose of vitamin D3 (a single enteral dose of 540,000 IU) supplementation had no advantage over placebo with respect to 90 day mortality (23.5% vs. 20.6%, *P* = 0.26) (5), providing further conflicting information on the effects of high-dose vitamin D3 in critically ill patients. Therefore, a quantitative analysis of the pooled results of up-to-date trials is required to validate the effects of high dose vitamin D3 on the prognosis of critically ill patients.

## Materials and Methods

This analysis was performed according to the Preferred Reporting Items for Systematic Reviews and Meta-Analyses (PRISMA) statement (15) (Supplemental File 1) and was registered in the International Prospective Register of Systematic Reviews (CRD42020179195).

### Search Strategy

We searched the following databases until December 4th, 2020, for appropriate articles: PubMed, Web of Science, EMBASE, Cochrane Central database. The following MeSH terms were used: “vitamin D3” “Cholecalciferol” “Critical Care” and “Intensive Care Unit”. The full search strategy is available in Supplemental File 2.

### Eligibility Criteria

We enrolled trials with the following PICOS criteria: (1) Population: adult patients (aged more than 18 years) who were admitted to the ICU; (2) Intervention: administration of high-dose vitamin D3 (a single dose from 300,000 IU to 540,000 IU), either enteral delivery or intramuscular injection; (3) Comparison intervention: placebo-control; (4) Outcome: mortality; (5) Study design: RCT. There was no language restriction. The exclusion criteria were duplicates or overlapping populations and lack of data on mortality.

### Study Selection and Data Extraction

Two researchers independently screened titles and abstracts in duplicate to determine whether a particular trial met the inclusion criteria. The full texts of potentially eligible trails were subject to an independent review process. To resolve discrepancies, we discussed with a third reviewer and contacted the study authors if necessary.

The primary outcome of the meta-analysis was mortality, including mortality truncated to day 28 and day 90. If the trail did not reported the 28 day or 90 day mortality, we used the ICU or hospital mortality or 30 day mortality instead. The secondary outcomes were ventilator days, length of ICU and hospital stay, and adverse events related to the interventions (hypercalcemia, hyperphosphatemia, fall and fall-related fracture, and the level of total and ionized calcemia, phosphorus and creatinine). If the continuous variables were reported as 95% confidence interval, they would be converted and described as the mean with standard deviation.

The Grading of Recommendations Assessment, Development and Evaluation approach was used to evaluate the quality of the evidence for outcomes (16).

### Risk of Bias Assessment

Cochrane Collaboration's protocols were used to evaluate the internal validity and risk of bias by two reviewers separately (17). We checked the procedures performed in the included RCTs, and evaluated the risk of bias as “yes”, “no” or “unclear”.

### Statistical Analysis

The data were extracted analyzed by Cochrane Collaboration software Revman 5.1 (The Nordic Cochrane Centre, Rigshospitalet, Copenhagen, Denmark). We used Mantel-Haneszel (M-H) chi-square and *I*^2^ tests to quantify the statistical heterogeneity and inconsistency of the included RCTs (18). *P* < 0.1 was defined as statistically significant heterogeneity for the M-H chi-square test. We used Cochrane *I*^2^ statistics to assess the heterogeneity, while *I*^2^ ≥ 50% was defined as high heterogeneity and the random-effects model would be used. Each study was sequentially removed, and we reanalyzed the remaining dataset for statistical significance. Univariate meta-regression was used to explore the potential sources of heterogeneity. And we used Post-hoc subgroup analysis to analyze the effects of vitamin D3 in critically ill patients. We tested for publication bias of the outcomes by Egger's test.

### Trial Sequential Analysis

TSA (TSA software version 0.9 Beta; Copenhagen Trial Unit, Copenhagen, Denmark) was used to adjust the threshold for statistical significance in the cumulative meta-analysis due to type I errors, which were caused by an increased risk of random error and repeated significance testing (19, 20). We calculated information size as the diversity-adjusted information size (DIS), which was suggested by the relative risk reduction (RRR) of the intervention in the included trials (20). We estimated 28% mortality in the placebo group and a reduction of mortality to 21% in the intervention group, adopted from the VITdAL-ICU study (8), with 80% power and a two-sided alpha of 0.05.

## Results

**Figure 1:**
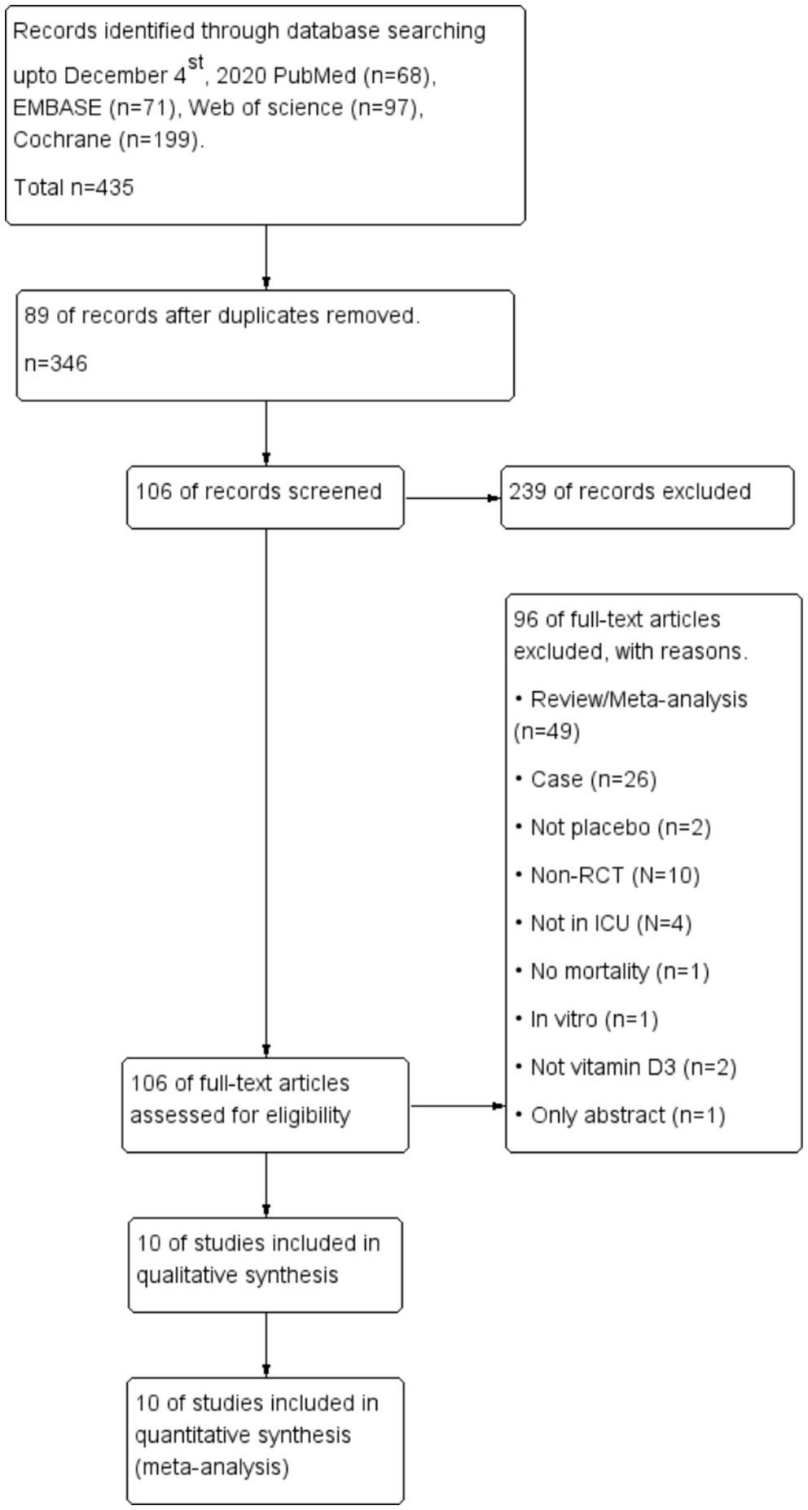
Flow chart of the search process and study selection.

The comprehensive search yielded a total of 435 articles, and 10 RCTs enrolling 2058 patients were finally included in the meta-analysis (5–9, 21–25) (Figure 1). Four of the 10 RCTs were conducted in the USA (5–7, 25), 2 in Austria (8, 23), 3 in Iran (9, 22, 24), and 1 in China (21). Three RCTs were designed as multicenter RCTs (5, 6, 9), and the others were single center RCTs (7, 8, 21–26). All the enrolled trial intervention groups received a high dose of vitamin D3, given orally or *via* nasogastric tube in 6 trials (5–8, 23, 25) and *via* intramuscular injection in the remaining 4 trails (9, 21, 22, 24) (Table 1).

**Table 1:** Baseline characteristics of included trails.

**Trial**	**Country**	**No. of centers**	**Sample size, n**	**Eligible patients**	**Vitamin D prescription**	**Baseline 25(OH)D** **level (ng/ml)**	
						**Vitamin D group**	**Placebo group**
Hasanloei et al. (24)	Iran	Single center	48	Adult patients with an expectedn need of mechanical ventialtion ≥48h and at least 7 days stay in ICU;10 ≤ 25(OH)D ≤ 30 ng/mL	Intromuscular cholecalciferol 300,000 IU	18.66 ± 3.28	**17** ±**3.25**
VIOLET (5)	USA	Multicenter, 44 hospitals	1,059	Adults and had one or more acute risk factors for death or lung injury needed for ICU admission.	A single enteral dose of 540,000 IU vitamin D3	11.2 ± 4.8	11.0 ± 4.7
Miri et al. (22)	Iran	Single center	40	adult (age between 18 and 65 years) patients with MV	Intromuscular vitamin D3 300,000 IU	8.43 ± 6.8	11.35 ± 18.23
Karsy et al. (25)	USA	Single center	267	age≥18 years, an expected ICU stay ≥48 h, 25(OH)D ≤ 20 ng/mL	A single dose of vitamin D3 540,000IU orally	14.6 ± 4.2	13.9 ± 4.6
Ding et al. (21)	China	Single center	57	ICU stay >48 h sepsis patients	Intromuscular vitamin D3 300,000 IU	–	–
Miroliaee et al. (9)	Iran	Multicenter, 2 hospitals	46	>18 years old who had been diagnosed with VAP	Intromuscular vitamin D3 300,000 IU	17.12 ± 6.11	19.5 ± 4.60
Han et al. (6)	USA	Multicenter, 2 hospitals	21	Receiving in ICU; ≥18 years; Expected to require MV≥72 hours; expected to remain in ICU ≥96 hours	2 pills of 50,000IU of vitamin D3 daily for 5 days	20.0 ± 7.3	21.5 ± 12.2
Quraishi et al. (3)	USA	Single center, 3 ICUs	20	≥18 years; within 24 h of new-onset sepsis	A single enteral dose of 400,000IU cholecalciferol	17 (13–25)	19 (13–22)
Amrein et al. (8)	Austria	Single center, 5 ICUs	475	≥18 years; expected to stay in ICU ≥48 h; 25(OH)D ≤ 20 ng/mL	Loading dose of 540,000 IU of vitamin D_3_ orally or *via* nasogastric tube	13.0 ± 4.1	13.1 (9.7–16.6)
Amrein et al. (23)	Austria	Single center	25	25(OH)D-deficient adult patients with expected ICU stay ≥48 h	540,000 IU of vitamin D3 orally or *via* feeding tube	13.1	14.1

*The bold value means that the serum 25(OH)D level was lower than normal values indicating that the patients was in vitamin D deficiency conditions*.

### Primary Outcomes

**Figure 2:**
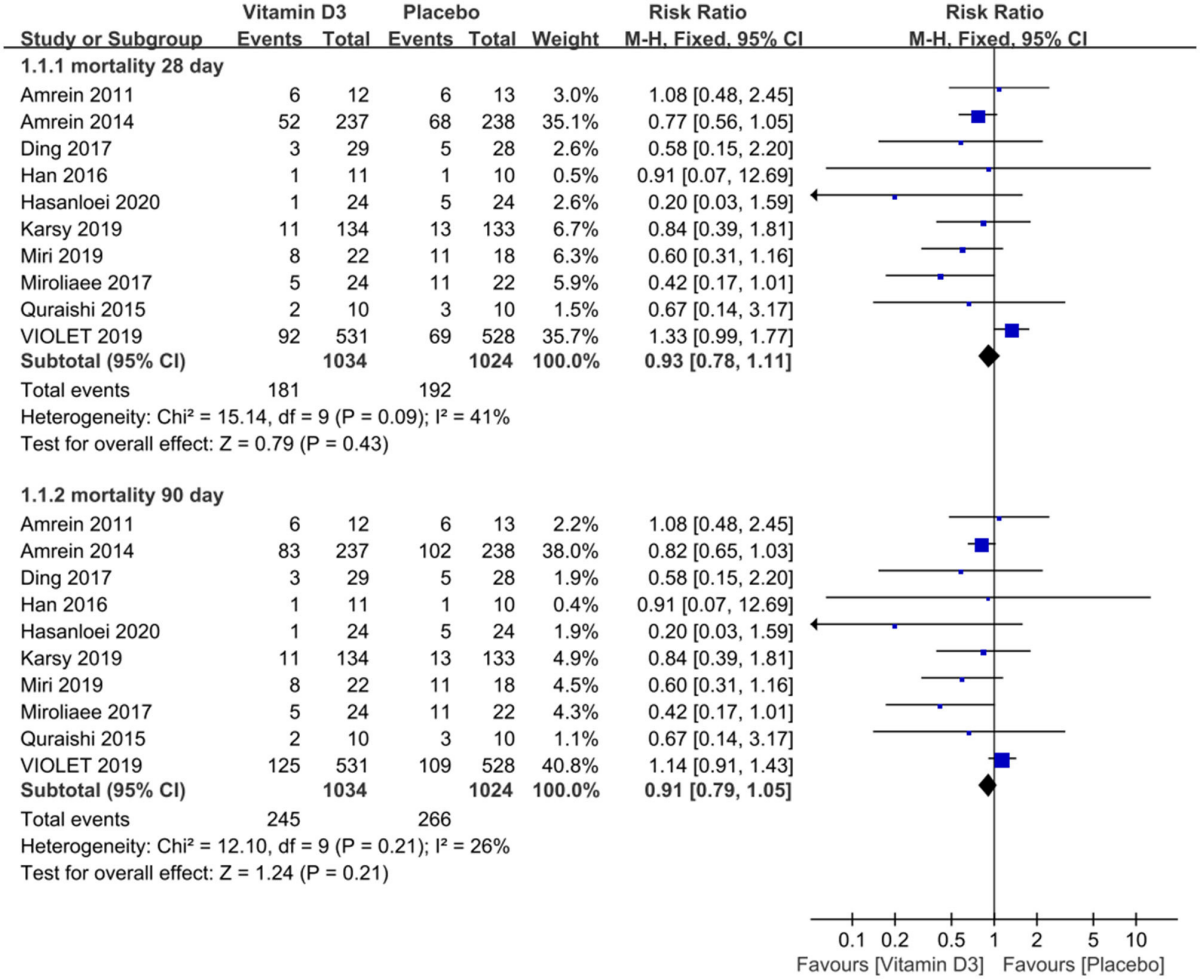
The effect of vitamin D3 on mortality truncated to day 28 and day 90 in critically ill adult patients.

The mortality data extracted from the included trials were pooled and analyzed, and the results revealed that compared with that of the placebo group, there was no significant decrease in mortality in the vitamin D3 group, with an RR of 0.93 (95% CI 0.78–1.11, *P* = 0.43), when the observation endpoint was truncated to day 28. Additionally, we did not observe a significant difference in mortality between the two groups, with an RR of 0.91 (95% CI 0.79–1.05, *P* = 0.21), when truncated to day 90 (Figure 2).

### Secondary Outcomes

**Figure 3:**
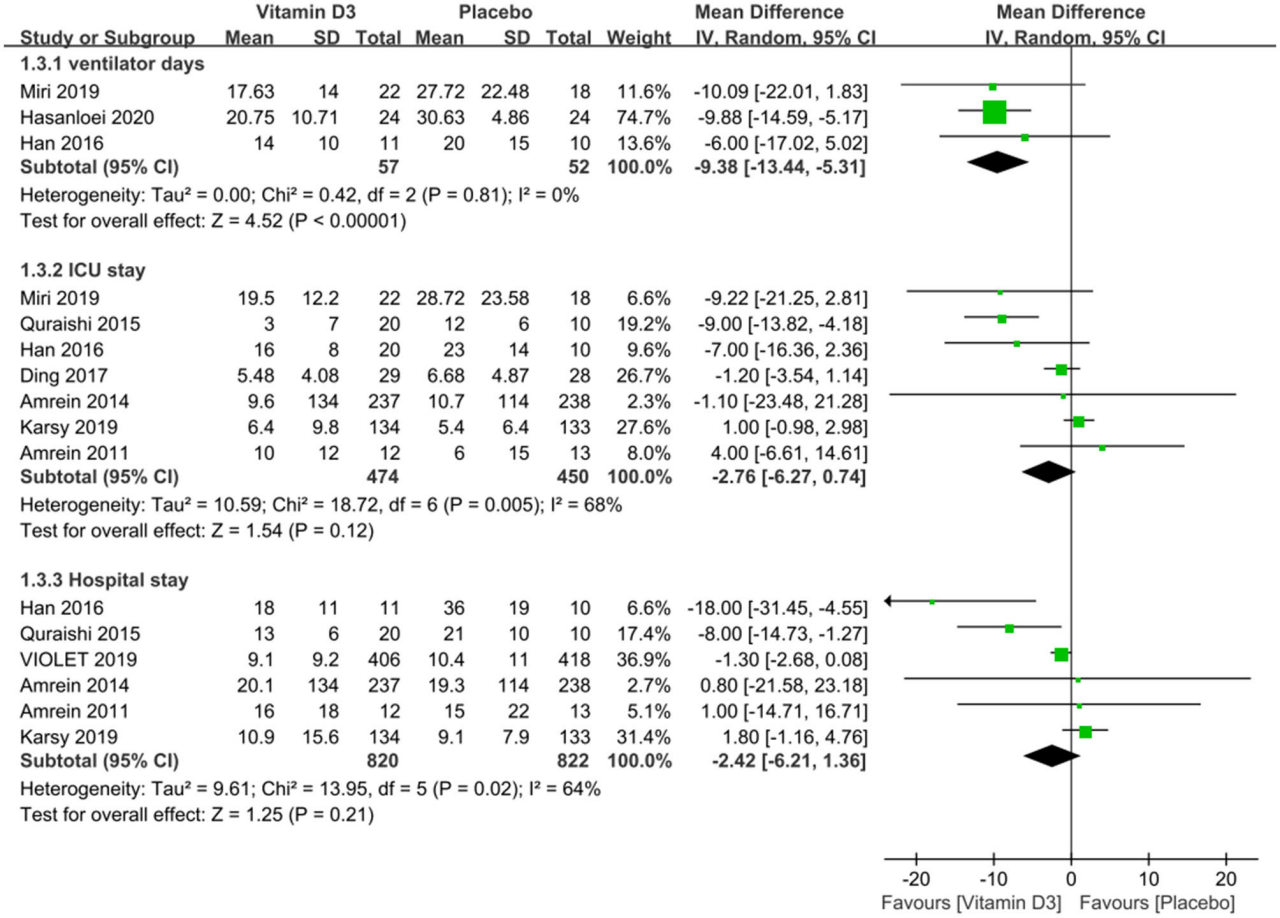
The effect of vitamin D3 on the length of ICU stay and hospital stay in critically ill adult patients.

We compared the ventilator days between the vitamin D3 and placebo groups and found that the use of vitamin D3 reduced the ventilator days (MD −9.38, 95%CI −13.44 to −5.31, *P* < 0.001), and also we compared the length of ICU and hospital stay and found that the length of ICU stay (MD −2.76, 95% CI −6.27 to 0.74, *P* = 0.12) and hospital stay (MD −2.42, 95% CI −6.21 to 1.36, *P* = 0.21) were similar between groups (Figure 3).

**Figure 4:**
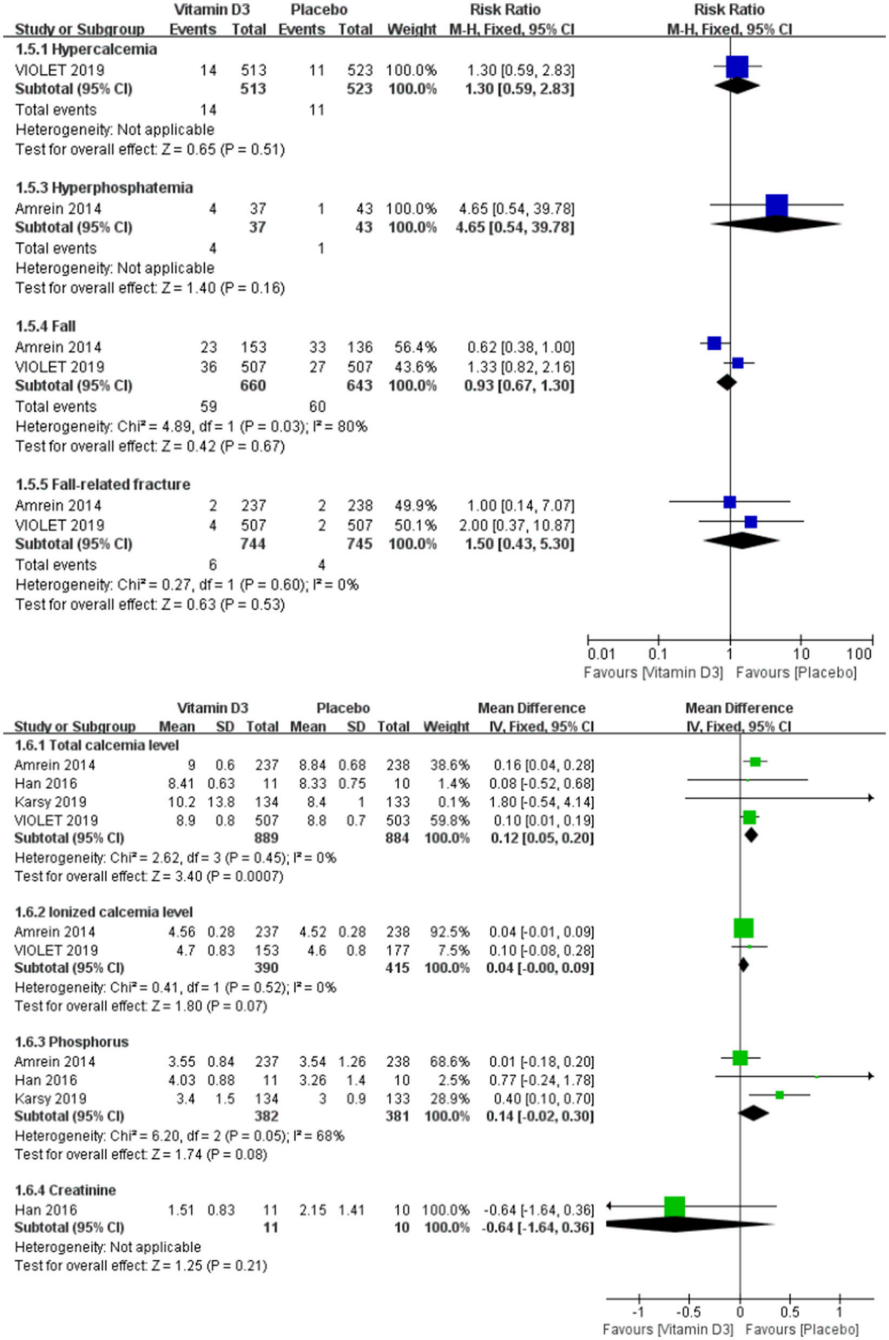
Forest plots of adverse events between the vitamin D3 group and the placebo group in critically ill adult patients.

No significant difference could be observed in the adverse events, including hypercalcemia (RR 1.30, 95% CI 0.59–2.83, *P* = 0.51), hyperphosphatemia (RR 4.65, 95% CI 0.54–39.78, *P* = 0.16), fall (RR 0.93, 95% CI 0.67–1.30, *P* = 0.67) and fall-related fracture (RR 1.50, 95% CI 0.43–5.30, *P* = 0.53). And there was no difference in the ionized calcium, phosphorus and creatinine level, except the total calcium level was significantly increase in vitamin D3 group (MD 0.12, 95% CI 0.05–0.20, *P* < 0.001) (Figure 4).

### Risk of Bias and Sensitivity Analysis

All included RCTs were evaluated for risk of bias items, including selection bias (random sequence generation and allocation concealment), performance bias (blinding of participants and personnel), detection bias (blinding of outcome assessment), attrition bias (incomplete outcome data), reporting bias (selective reporting) and other bias (Supplemental File 3). The detailed risk bias assessment of the included trials is provided in Supplemental File 4.

Egger's test for publication bias showed that there was no significant difference in the primary outcomes (mortality truncated to day 28, *P* = 0.100 [*t* = −1.86, *95% CI*: −2.57~0.28]); mortality truncated to day 90, *P* = 0.095 [*t* = −1.90, *95% CI*: −2.05~0.20]) (Supplemental File 5). Considering at least 10 trails are recommended when assessing publication bias by Egger's test (27), our meta meets the above condition. The Egger's test result indicates no publication bias.

Each trial was sequentially omitted to analyze the individual effects of the trial on the overall results, showing that there was a significant difference between groups when the VIOLET study (5) was omitted from the pooled analysis (Supplemental File 6).

Variable risks of bias were analyzed in all included trials to downgrade the quality of the evidence. The GRADE levels of evidence for the mortality truncated to day 28 and for the mortality truncated to day 90 were both low (Supplemental File 7).

### Trial Sequential Analysis

TSA indicated that the current information size did not cross the Lan-DeMets sequential monitoring boundary by the optimal information size, suggesting insufficient sample size in investigating the mortality truncated to day 28. An optimal sample size of 2,158 patients was estimated, which was expected to reach the plausible endpoint (Supplemental File 8).

### Meta-Regression and Subgroup Analysis

Univariate meta-regression revealed that the sample size (*P* = 0.012), vitamin D3 dosage (*P* = 0.039) and the method of administration (*P* = 0.041) might be associated with the heterogeneity between studies. The full list of factors involved in the univariate meta-regression is provided in Supplemental File 9. A post hoc subgroup analysis based on the dosage and administration route of vitamin D3 was performed and found that the mortality truncated to day 28 might significantly decrease in patients who received vitamin D3 300,000 IU, with an RR of 0.47 (95% CI 0.29–0.77, *P* = 0.003) (Supplemental File 10), and in patients who were intramuscularly administered, with an RR of 0.47 (95% CI 0.29–0.77, *P* = 0.003) (Supplemental File 11).

## Discussion

In the present meta-analysis, we pooled the results from 10 RCTs on the use of a high dose of vitamin D3 in critically ill adult patients and found that the high dose of vitamin D3 did not reduce mortality truncated to day 28 and day 90, but was associated with decreased length of ventilator days. No statistic differences were found in the length of ICU and hospital stay.

Our results seemed at odds with a previous meta-analysis that indicated that vitamin D3 administration was associated with a significant reduction in mortality at the longest follow-up available (14). Our sensitivity analysis suggested that removal of the VIOLET study caused substantial changes in the final results, suggesting that the VIOLET trial was the main reason for the difference between our results and the previous meta-analysis (14). The VIOLET trial confirmed that high-dose vitamin D3 did not reduce the mortality at day 28 and day 90. Some limitations need attention in the VIOLET study, including that the study included mild critically ill patients (total SOFA score in the vitamin D3 group was 5.6 .ou.6 and 5.4 an.7 in the placebo group); the 25[OH]D level of included patients was <20 ng/ml rather than 12 ng/ml, who were more likely to benefit from vitamin D supplementation; 23.6% of the vitamin D3 group patient's 25[OH]D level were still lower than 30 ng/ml at day 3; and also the lack of maintenance doses of vitamin D3, which were all likely to bias the trial to null (28). The ongoing VITDALIZE study, including ICU patients with 25[OH]D level <12 ng/ml who received a bolus of 540,000 IU vitamin D3 followed by 4,000 IU daily for 90 days will advance our knowledge in this field (29).

High dose of vitamin D3 did not improve clinical outcomes in critically ill patients (30). There might be several potential explanations. First, vitamin D3 supplementation is widely practiced in westernized populations (31), which might dilute the effect of high dose provided during RCTs. Second, vitamin D3 supplementations in RCTs were provided as inactive form that need ongoing metabolic steps to be activated. However, many critically ill patients are seemed to be incapable of activating native vitamin D sufficiently (8). Third, high dose of vitamin D3 supplementation may not be sufficient to fill the stocks and fill the pre-existing deficit in critically ill patients (5, 8). Lack of effect might be due to the failure to restore adequate status following the supplementation. Fourth, vitamin D3 supplementation in RCTs was limited in time and did not reflect chronic impregnation of the body. And last, vitamin D3 was given in supra physiological dose, alone without synergistic factors (32, 33), which could inhibit related metabolic pathways. In severe acute illness, the optimal vitamin D3 dosage remains unclear. Rapid decreases in circulating 25[OH]D concentration were proven to be highly prevalent in critical illness (34, 35). Therefore, the use of a high loading dose for the rapid restoration of vitamin D levels appears necessary (36). However, various high loading doses (vitamin D3, 300,000–540,000 IU) were employed in the current studies. No standard for the high dose has been established. A significantly decrease mortality was observed in the subgroup of critically ill patients with vitamin D3 300,000IU. And it also appears reasonable that 540,000 IU, which has been proven to be safe and effective (5, 8), could be administered in critical illness (11).

Interestingly, we observed that there was a significant decrease in mortality in the subgroup of patients whose vitamin D3 was administered by intramuscular injection. Whyte MP et al. proved that compared with oral or iv dosing, intramuscular injections of vitamin D resulted in prolonged increased serum 25[OH]D level (37). Given the prevalence of gastrointestinal dysfunction and the unreliability of enteral absorption in the critically ill population (38), intramuscular supplementation may be a more effective alternative for vitamin D repletion (26). Due to the limited sample size, we are cautious about the improved prognosis.

We found that the ventilator days were significantly decreased after high dose vitamin D3 supplementation. Some trials have revealed the molecular role of vitamin D3 in skeletal muscle tissue function and metabolism, such as suppressing inflammatory cytokines (39), decreasing the pulmonary vascular permeability index in high-risk lung injury patients (10), improving lung function (40), and positively correlating with muscle strength (12). These probably could explain why high dose vitamin D3 shorten the duration of mechanical ventilation.

Our study had several limitations. First, most of the trials included in our meta-analysis had a small sample size, while only one multicenter large-scale RCT was included. More trials are needed to further validate the effects of vitamin D3 in critically ill patients. Second, only two of the included RCTs in our meta-analysis adopted liquid chromatography-tandem mass spectrometry (LC-MS/MS), which is the reference method used to measure 25[OH]D levels. The other available methodologies showed a variable systematic bias in measured 25[OH]D values vs. LC-MS/MS (41). The analysis of mixed 25[OH]D measurements might have introduced bias to the final results. Third, we substituted other mortality rates for the 28 day and 90 day mortality, and the subgroup analyses included a relatively small number of studies, which could have potentially introduced bias and should be interpreted cautiously.

## Conclusions

A high dose of vitamin D3 was not associated with decreased mortality truncated to day 28 and day 90 in critically ill patients, but could significantly reduce the ventilator days. However, more large-scale RCTs are needed to further validate the effects of high dose vitamin D3 in critically ill patients.

## Data Availability Statement

The original contributions presented in the study are included in the article/Supplementary Material, further inquiries can be directed to the corresponding author/s.

## Author Contributions

ZG: conceptualization, methodology, formal analysis, writing—original draft, and supervision. JX: methodology and formal analysis. CL: conceptualization and validation. LL: formal analysis and writing—original draft. YY: methodology, formal analysis, writing—review and editing, and supervision. All authors contributed to the article and approved the submitted version.

## Funding

This work was supported by National Natural Science Foundation of China (81971888 and 81971829), Jiangsu Provincial Special Program of Medical Science (BE2018743), and Huai'an Health Research Project 2019 (HAWJ201905).

## Conflict of Interest

The authors declare that the research was conducted in the absence of any commercial or financial relationships that could be construed as a potential conflict of interest.

## Publisher's Note

All claims expressed in this article are solely those of the authors and do not necessarily represent those of their affiliated organizations, or those of the publisher, the editors and the reviewers. Any product that may be evaluated in this article, or claim that may be made by its manufacturer, is not guaranteed or endorsed by the publisher.

## Supplementary Material

Supplemental File 1
Checklist for the meta-analysis applied to this manuscript according to the Preferred Reporting Items for Systematic Reviews and Meta-Analysis (PRISMA) statement.

Supplemental File 2
Full search strategy for PubMed, Web of Science, EMBASE, and the Cochrane Central database.

Supplemental File 3
The risk of bias of the included trials.

Supplemental File 4
Risk of bias assessment of the included trials.

Supplemental File 5
Publication bias of mortality truncated to day 28 and day 90.

Supplemental File 6
Sensitivity analysis.

Supplemental File 7
GRADE profile for assessing the quality of evidence for vitamin D3 in critically ill patients.

Supplemental File 8
Trial sequential analysis revealing the optimal sample size for detecting the plausible effect of vitamin D3 use on mortality truncated to 28 days.

Supplemental File 9
Univariate meta-regression analysis.

Supplemental File 10
Subgroup analysis. Patients were divided by dose of vitamin D3 (300,000 IU, 400,000 IU, and 540,000 IU).

Supplemental File 11
Subgroup analysis. Patients were divided by vitamin D3 administration route (enteral and intramuscular).

## References

[1] Lucidarme O Messai E Mazzoni T Arcade M du Cheyron D. Incidence and risk factors of vitamin D deficiency in critically ill patients: results from a prospective observational study. Intensive Care Med. (2010) 36:1609–11. 10.1007/s00134-010-1875-8 20373095

[2] Vassiliou A G Jahaj E Mastora Z Stagaki E Orfanos S E Kotanidou A. Serum admission 25-hydroxyvitamin D levels and outcomes in initially non-septic critically Ill patients. Shock. (2018) 50:511–8. 10.1097/SHK.0000000000001105 29337839

[3] Quraishi S A McCarthy C Blum L Cobb J P Camargo C A. Plasma 25-Hydroxyvitamin D Levels at Initiation of Care and Duration of Mechanical Ventilation in Critically Ill Surgical Patients. JPEN J Parenter Enteral Nutr. (2016) 40:273–8. 10.1177/0148607114566276 25564428 PMC4492899

[4] Gomes T L Fernandes R C Vieira L L Schincaglia R M Mota J F Nobrega M S . Low vitamin D at ICU admission is associated with cancer, infections, acute respiratory insufficiency, and liver failure. Nutrition. (2019) 60:235–40. 10.1016/j.nut.2018.10.018 30682545

[5] National Heart L Blood Institute P C T N Ginde A A Brower R G Caterino J M Finck L. Early high-dose vitamin D3 for critically Ill, vitamin D-deficient patients. N Engl J Med. (2019) 381:2529–40. 10.1056/NEJMoa1911124 31826336 PMC7306117

[6] Han J E Jones J L Tangpricha V Brown M A Brown L A S Hao L . High dose vitamin D administration in ventilated intensive care unit patients: a pilot double blind randomized controlled trial. J Clin Transl Endocrinol. (2016) 4:59–65. 10.1016/j.jcte.2016.04.004 27419080 PMC4939707

[7] Quraishi S A De Pascale G Needleman J S Nakazawa H Kaneki M Bajwa E K . Effect of cholecalciferol supplementation on vitamin D status and cathelicidin levels in sepsis: a randomized, placebo-controlled trial. Crit Care Med. (2015) 43:1928–37. 10.1097/CCM.0000000000001148 26086941 PMC4537665

[8] Amrein K Schnedl C Holl A Riedl R Christopher K B Pachler C . Effect of high-dose vitamin D3 on hospital length of stay in critically ill patients with vitamin D deficiency: the VITdAL-ICU randomized clinical trial. JAMA. (2014) 312:1520–30. 10.1001/jama.2014.13204 25268295

[9] Miroliaee A E Salamzadeh J Shokouhi S Fatemi A Ardehali S H Hajiesmaeili M R . Effect of vitamin D supplementation on procalcitonin as prognostic biomarker in patients with ventilator associated pneumonia complicated with vitamin D deficiency. Iran J Pharm Res. (2017) 16:1254–63. 29201115 PMC5610782

[10] Parekh D Dancer R C A Scott A D'Souza V K Howells P A Mahida R Y . Vitamin D to prevent lung injury following esophagectomy-a randomized, placebo-controlled trial. Crit Care Med. (2018) 46:e1128–35. 10.1097/CCM.0000000000003405 30222631 PMC6250246

[11] Amrein K Papinutti A Mathew E Vila G Parekh D. Vitamin D and critical illness: what endocrinology can learn from intensive care and vice versa. Endocr Connect. (2018) 7:R304–15. 10.1530/EC-18-0184 30352414 PMC6240147

[12] Borges R C Barbeiro H V Barbeiro D F Soriano F G. Muscle degradation, vitamin D and systemic inflammation in hospitalized septic patients. J Crit Care. (2020) 56:125–31. 10.1016/j.jcrc.2019.12.017 31896446

[13] Martucci G McNally D Parekh D Zajic P Tuzzolino F Arcadipane A . Trying to identify who may benefit most from future vitamin D intervention trials: a post hoc analysis from the VITDAL-ICU study excluding the early deaths. Crit Care. (2019) 23:200. 10.1186/s13054-019-2472-z 31164148 PMC6549317

[14] Putzu A Belletti A Cassina T Clivio S Monti G Zangrillo A . Vitamin D and outcomes in adult critically ill patients. A systematic review and meta-analysis of randomized trials. J Crit Care. (2017) 38:109–14. 10.1016/j.jcrc.2016.10.029 27883968

[15] Liberati A Altman D G Tetzlaff J Mulrow C Gotzsche P C Ioannidis J P . The PRISMA statement for reporting systematic reviews and meta-analyses of studies that evaluate healthcare interventions: explanation and elaboration. BMJ. (2009) 339:b2700. 10.1136/bmj.b2700 19622552 PMC2714672

[16] Cumpston M Li T Page M J Chandler J Welch V A Higgins J P . Updated guidance for trusted systematic reviews: a new edition of the Cochrane Handbook for Systematic Reviews of Interventions. Cochrane Database Syst Rev. (2019) 10:ED000142. 10.1002/14651858.ED000142 31643080 PMC10284251

[17] Higgins J P Altman D G Gotzsche P C Juni P Moher D Oxman A D . The Cochrane Collaboration's tool for assessing risk of bias in randomised trials. BMJ. (2011) 343:d5928. 10.1136/bmj.d5928 22008217 PMC3196245

[18] Higgins J P Thompson S G Deeks J J Altman D G. Measuring inconsistency in meta-analyses. BMJ. (2003) 327:557–60. 10.1136/bmj.327.7414.557 12958120 PMC192859

[19] Brok J Thorlund K Gluud C Wetterslev J. Trial sequential analysis reveals insufficient information size and potentially false positive results in many meta-analyses. J Clin Epidemiol. (2008) 61:763–9. 10.1016/j.jclinepi.2007.10.007 18411040

[20] Wetterslev J Thorlund K Brok J Gluud C. Estimating required information size by quantifying diversity in random-effects model meta-analyses. BMC Med Res Methodol. (2009) 9:86. 10.1186/1471-2288-9-86 20042080 PMC2809074

[21] Ding F Zang B Fu J Ji K. Effect of vitamin D3 on the severity and prognosis of patients with sepsis: a prospective randomized double-blind placebo study. Zhonghua Wei Zhong Bing Ji Jiu Yi Xue. (2017) 29:106–10. 28625255 10.3760/cma.j.issn.2095-4352.2017.02.003

[22] Miri M Kouchek M Rahat Dahmardeh A Sistanizad M. Effect of High-Dose Vitamin D on Duration of Mechanical Ventilation in ICU Patients. Iran J Pharmaceut Res IJPR. (2019) 18:1067–72. 31531088 10.22037/ijpr.2019.1100647 PMC6706749

[23] Amrein K Sourij H Wagner G Holl A Pieber T R Smolle K H . Short-term effects of high-dose oral vitamin D3 in critically ill vitamin D deficient patients: a randomized, double-blind, placebo-controlled pilot study. Crit Care. (2011) 15:R104. 10.1186/cc10120 21443793 PMC3219377

[24] Hasanloei M A V Rahimlou M Eivazloo A Sane S Ayremlou P Hashemi R. Effect of oral versus intramuscular vitamin D replacement on oxidative stress and outcomes in traumatic mechanical ventilated patients admitted to intensive care unit. Nutr Clin Pract. (2020) 35:548–58. 10.1002/ncp.10404 31486158

[25] Karsy M Guan J Eli I Brock AA Menacho ST Park MS. The effect of supplementation of vitamin D in neurocritical care patients: RandomizEd Clinical TrIal oF hYpovitaminosis D (RECTIFY). J Neurosurg. (2019) 1–10. 10.3171/2018.11.JNS182713 31518978

[26] Nair P Venkatesh B Lee P Kerr S Hoechter D J Dimeski G . A Randomized study of a single dose of intramuscular cholecalciferol in critically Ill adults. Crit Care Med. (2015) 43:2313–20. 10.1097/CCM.0000000000001201 26186566

[27] Egger M Davey Smith G Schneider M Minder C. Bias in meta-analysis detected by a simple, graphical test. BMJ. (1997) 315:629–34. 10.1136/bmj.315.7109.629 9310563 PMC2127453

[28] Preiser J C Christopher K. High-dose vitamin D3 for critically Ill vitamin D-deficient patients. N Engl J Med. (2020) 382:1670. 10.1056/NEJMc2000993 32320584

[29] Amrein K Parekh D Westphal S Preiser J C Berghold A Riedl R . Effect of high-dose vitamin D3 on 28-day mortality in adult critically ill patients with severe vitamin D deficiency: a study protocol of a multicentre, placebo-controlled double-blind phase III RCT (the VITDALIZE study). BMJ Open. (2019) 9:e031083. 10.1136/bmjopen-2019-031083 31722941 PMC6858186

[30] Langlois P L Szwec C D'Aragon F Heyland D K Manzanares W. Vitamin D supplementation in the critically ill: A systematic review and meta-analysis. Clin Nutr. (2018) 37:1238–46. 10.1016/j.clnu.2017.05.006 28549527

[31] Ross A C. The 2011 report on dietary reference intakes for calcium and vitamin D. Public Health Nutr. (2011) 14:938–9. 10.1017/S1368980011000565 21492489

[32] Zhang V Jiang X Farukhi Y Z Rosen C J Schnatz P F. Vitamin D and calcium: what do we need to know? Clin Obstet Gynecol. (2013) 56:654–8. 10.1097/GRF.0b013e3182a98274 24047935

[33] Barrea L Muscogiuri G Frias-Toral E Laudisio D Pugliese G Castellucci B . Nutrition and immune system: from the Mediterranean diet to dietary supplementary through the microbiota. Crit Rev Food Sci Nutr. (2021) 61:3066–90. 10.1080/10408398.2020.1792826 32691606

[34] De Pascale G Quraishi S A Vitamin D status in critically ill patients: the evidence is now bioavailable! Crit Care. (2014) 18:449. 10.1186/cc13975 25043906 PMC4095727

[35] Czarnik T Czarnik A Gawda R Gawor M Piwoda M Marszalski M . Vitamin D kinetics in the acute phase of critical illness: a prospective observational study. J Crit Care. (2018) 43:294–9. 10.1016/j.jcrc.2017.09.179 28968524

[36] McNally J D. Vitamin D as a modifiable risk factor in critical illness: questions and answers provided by observational studies. J Pediatr (Rio J). (2014) 90:99–101. 10.1016/j.jped.2013.12.002 24361295

[37] Whyte MP Haddad JG Walters DD Stamp TC. Vitamin D bioavailability: serum 25-hydroxyvitamin D levels in man after oral, subcutaneous, intramuscular, and intravenous vitamin D administration. J Clin Endocrinol Metab. (1979) 48:906–11. 10.1210/jcem-48-6-906 447796

[38] Reintam Blaser A Poeze M Malbrain M L Bjorck M. Oudemans-van Straaten H M, Starkopf J, et al. Gastrointestinal symptoms during the first week of intensive care are associated with poor outcome: a prospective multicentre study. Intensive Care Med. (2013) 39:899–909. 10.1007/s00134-013-2831-1 23370829 PMC3625421

[39] Van den Berghe G Van Roosbroeck D Vanhove P Wouters P J De Pourcq L Bouillon R. Bone turnover in prolonged critical illness: effect of vitamin D. J Clin Endocrinol Metab. (2003) 88:4623–32. 10.1210/jc.2003-030358 14557432

[40] Sutherland E R Goleva E Jackson L P Stevens A D Leung D Y. Vitamin D levels, lung function, and steroid response in adult asthma. Am J Respir Crit Care Med. (2010) 181:699–704. 10.1164/rccm.200911-1710OC 20075384 PMC2868500

[41] Roth H J Schmidt-Gayk H Weber H Niederau C. Accuracy and clinical implications of seven 25-hydroxyvitamin D methods compared with liquid chromatography-tandem mass spectrometry as a reference. Ann Clin Biochem. (2008) 45:153–9. 10.1258/acb.2007.007091 18325178

