# Supplementary material for: High Dose Vitamin D3 Supplementation Is Not Associated With Lower Mortality in Critically Ill Patients: A Meta-Analysis of Randomized Control Trials
Source: Front Nutr. 2022 May 4;9:762316. doi: 10.3389/fnut.2022.762316 (PMC9116294; doi:10.3389/fnut.2022.762316)
Supplement: Supplemental File 2 — Full search strategy for PubMed, Web of Science, EMBASE, and the Cochrane Central database. [file Image_2.pdf]

pubmed
#1 "Cholecalciferol"[Mesh]
#2 ((((Calciol[Title/Abstract]) OR ((3 beta,5Z,7E)-9,10-Secocholesta-5,7,10(19)-trien-3-ol[Title/Abstract])) OR (Vitamin D 3[Title/Abstract])) OR (Vitamin D3[Title/Abstract])) OR (Cholecalciferols[Title/Abstract])
#3 #1 AND #2 ("Cholecalciferol"[Mesh]) OR (((((Calciol[Title/Abstract]) OR ((3 beta,5Z,7E)-9,10-Secocholesta-5,7,10(19)-trien-3-ol[Title/Abstract])) OR (Vitamin D 3[Title/Abstract])) OR (Vitamin D3[Title/Abstract])) OR (Cholecalciferols[Title/Abstract]))

#4 "Critical Care"[Mesh]
#5 (((((Care, Critical[Title/Abstract]) OR (Intensive Care[Title/Abstract])) OR (Care, Intensive[Title/Abstract])) OR (Surgical Intensive Care[Title/Abstract])) OR (Care, Surgical Intensive[Title/Abstract])) OR (Intensive Care, Surgical[Title/Abstract])
#6 #4 AND #5 ("Critical Care"[Mesh]) OR ((((((Care, Critical[Title/Abstract]) OR (Intensive Care[Title/Abstract])) OR (Care, Intensive[Title/Abstract])) OR (Surgical Intensive Care[Title/Abstract])) OR (Care, Surgical Intensive[Title/Abstract])) OR (Intensive Care, Surgical[Title/Abstract]))

#7 Intensive Care Unit[MeSH Terms]
#8 ((((Care Unit, Intensive[Title/Abstract]) OR (Care Units, Intensive[Title/Abstract])) OR (Intensive Care Unit[Title/Abstract])) OR (Unit, Intensive Care[Title/Abstract])) OR (Units, Intensive Care[Title/Abstract])
#9 #7 AND #8 (Intensive Care Unit[MeSH Terms]) OR (((((Care Unit, Intensive[Title/Abstract]) OR (Care Units, Intensive[Title/Abstract])) OR (Intensive Care Unit[Title/Abstract])) OR (Unit, Intensive Care[Title/Abstract])) OR (Units, Intensive Care[Title/Abstract]))

#10 #6 OR #9 ((Intensive Care Unit[MeSH Terms]) OR (((((Care Unit, Intensive[Title/Abstract]) OR (Care Units, Intensive[Title/Abstract])) OR (Intensive Care Unit[Title/Abstract])) OR (Unit, Intensive Care[Title/Abstract])) OR (Units, Intensive Care[Title/Abstract]))) OR (("Critical Care"[Mesh]) OR ((((((Care, Critical[Title/Abstract]) OR (Intensive Care[Title/Abstract])) OR (Care, Intensive[Title/Abstract])) OR (Surgical Intensive Care[Title/Abstract])) OR (Care, Surgical Intensive[Title/Abstract])) OR (Intensive Care, Surgical[Title/Abstract])))

#11 #3 AND #10 (((Intensive Care Unit[MeSH Terms]) OR (((((Care Unit, Intensive[Title/Abstract]) OR (Care Units, Intensive[Title/Abstract])) OR (Intensive Care Unit[Title/Abstract])) OR (Unit, Intensive Care[Title/Abstract])) OR (Units, Intensive Care[Title/Abstract]))) OR (("Critical Care"[Mesh]) OR ((((((Care, Critical[Title/Abstract]) OR (Intensive Care[Title/Abstract])) OR (Care, Intensive[Title/Abstract])) OR (Surgical Intensive Care[Title/Abstract])) OR (Care, Surgical Intensive[Title/Abstract])) OR (Intensive Care, Surgical[Title/Abstract])))) AND (("Cholecalciferol"[Mesh]) OR (((((Calciol[Title/Abstract]) OR ((3 beta,5Z,7E)-9,10-Secocholesta-5,7,10(19)-trien-3-ol[Title/Abstract])) OR (Vitamin D 3[Title/Abstract])) OR (Vitamin D3[Title/Abstract])) OR (Cholecalciferols[Title/Abstract])))

#12 (clinical[tiab] AND trial[tiab]) OR "clinical trials as topic"[mesh] OR "clinical trial"[pt] OR

random*[tiab] OR "random allocation"[mesh] OR "therapeutic use"[sh]

#13 #11 AND #12 ((((Intensive Care Unit[MeSH Terms]) OR (((((Care Unit, Intensive[Title/Abstract]) OR (Care Units, Intensive[Title/Abstract])) OR (Intensive Care Unit[Title/Abstract])) OR (Unit, Intensive Care[Title/Abstract])) OR (Units, Intensive Care[Title/Abstract]))) OR (("Critical Care"[Mesh]) OR ((((((Care, Critical[Title/Abstract]) OR (Intensive Care[Title/Abstract])) OR (Care, Intensive[Title/Abstract])) OR (Surgical Intensive Care[Title/Abstract])) OR (Care, Surgical Intensive[Title/Abstract])) OR (Intensive Care, Surgical[Title/Abstract])))) AND (("Cholecalciferol"[Mesh]) OR (((((Calciol[Title/Abstract]) OR ((3 beta,5Z,7E)-9,10-Secocholesta-5,7,10(19)-trien-3-ol[Title/Abstract])) OR (Vitamin D 3[Title/Abstract])) OR (Vitamin D3[Title/Abstract])) OR (Cholecalciferols[Title/Abstract])))) AND ((clinical[tiab] AND trial[tiab]) OR "clinical trials as topic"[mesh] OR "clinical trial"[pt] OR random*[tiab] OR "random allocation"[mesh] OR "therapeutic use"[sh])

Web of science

#1	TS=(Cholecalciferol OR Calciol OR (3 beta,5Z,7E) -9,10-Secocholesta-5,7,10(19)-trien-3-ol OR Vitamin D 3 OR Vitamin D3 OR Cholecalciferols)<br>*Databases= WOS, BCI, CSCD, DIIDW, KJD, MEDLINE, RSCI, SCIELO Timespan=1900-2020*<br>*Search language=Auto*
#2	TS=(Intensive Care Units OR Care Unit, Intensive OR Care Units, Intensive OR Intensive Care Unit OR Unit, Intensive Care OR Units, Intensive Care)<br>*Databases= WOS, BCI, CSCD, DIIDW, KJD, MEDLINE, RSCI, SCIELO Timespan=1900-2020*<br>*Search language=Auto*
#3	TS=(Critical care OR Care, Critical OR Intensive Care OR Care, Intensive OR Surgical Intensive Care OR Care, Surgical Intensive OR Intensive Care, Surgical)<br>*Databases= WOS, BCI, CSCD, DIIDW, KJD, MEDLINE, RSCI, SCIELO Timespan=1900-2020*<br>*Search language=Auto*
#4	TS=(randomized controlled trial OR random)<br>*Databases= WOS, BCI, CSCD, DIIDW, KJD, MEDLINE, RSCI, SCIELO Timespan=1900-2020*<br>*Search language=Auto*
#5	#2 OR #3
#6	#1 AND #4 AND #5

EMBASE

#1 'colecalciferol'/exp OR calciol:ab,ti OR '(3 beta,5z,7e)-9,10-secocholesta-5,7,10(19)-trien-3-ol':ab,ti OR 'vitamin d 3':ab,ti OR 'vitamin

d3':ab,ti OR cholecalciferols:ab,ti
#2 'intensive care'/exp OR 'care, critical':ab,ti OR 'intensive care':ab,ti OR 'care, intensive':ab,ti OR 'surgical intensive care':ab,ti OR 'intensive care, surgical':ab,ti
#3 'intensive care unit'/exp OR 'burn unit':ab,ti OR 'coronary care unit':ab,ti OR 'medical intensive care unit':ab,ti OR 'neonatal intensive care unit':ab,ti OR 'neurological intensive care unit':ab,ti OR 'pediatric intensive care unit':ab,ti OR 'psychiatric intensive care unit':ab,ti OR 'stroke unit':ab,ti OR 'surgical intensive care unit':ab,ti
#4 #2 OR #3
#5 #4 AND #1
('colecalciferol'/exp OR calciol:ab,ti OR '(3 beta,5z,7e)-9,10-secocholesta-5,7,10(19)-trien-3-ol':ab,ti OR 'vitamin d 3':ab,ti OR 'vitamin d3':ab,ti OR cholecalciferols:ab,ti) AND ('intensive care'/exp OR 'care, critical':ab,ti OR 'intensive care':ab,ti OR 'care, intensive':ab,ti OR 'surgical intensive care':ab,ti OR 'intensive care, surgical':ab,ti OR 'intensive care unit'/exp OR 'burn unit':ab,ti OR 'coronary care unit':ab,ti OR 'medical intensive care unit':ab,ti OR 'neonatal intensive care unit':ab,ti OR 'neurological intensive care unit':ab,ti OR 'pediatric intensive care unit':ab,ti OR 'psychiatric intensive care unit':ab,ti OR 'stroke unit':ab,ti OR 'surgical intensive care unit':ab,ti)
#6 'randomized controlled trial'/exp
#7 #6 AND #5
('colecalciferol'/exp OR calciol:ab,ti OR '(3 beta,5z,7e)-9,10-secocholesta-5,7,10(19)-trien-3-ol':ab,ti OR 'vitamin d 3':ab,ti OR 'vitamin d3':ab,ti OR cholecalciferols:ab,ti) AND ('intensive care'/exp OR 'care, critical':ab,ti OR 'intensive care':ab,ti OR 'care, intensive':ab,ti OR 'surgical intensive care':ab,ti OR 'intensive care, surgical':ab,ti OR 'intensive care unit'/exp OR 'burn unit':ab,ti OR 'coronary care unit':ab,ti OR 'medical intensive care unit':ab,ti OR 'neonatal intensive care unit':ab,ti OR 'neurological intensive care unit':ab,ti OR 'pediatric intensive care unit':ab,ti OR 'psychiatric intensive care unit':ab,ti OR 'stroke unit':ab,ti OR 'surgical intensive care unit':ab,ti) AND 'randomized controlled trial'/exp

Cochrane
#1 MeSH descriptor: [Cholecalciferol] explode all trees
#2 (Calciol):ti,ab,kw
#3 ((3 beta,5Z,7E)-9,10-Secocholesta-5,7,10(19)-trien-3-ol):ti,ab,kw (ERROR)
#4 (Vitamin D 3):ti,ab,kw
#5 (Vitamin D3):ti,ab,kw
#6 (Cholecalciferols):ti,ab,kw
#7 #1 OR #2 OR #3 OR #4 OR #5 OR #6
#8 MeSH descriptor: [Critical Care] explode all trees
#9 (Care, Critical):ti,ab,kw
#10 (Intensive Care):ti,ab,kw
#11 (Care, Intensive):ti,ab,kw
#12 (Surgical Intensive Care):ti,ab,kw
#13 (Care, Surgical Intensive):ti,ab,kw
#14 (Intensive Care, Surgical):ti,ab,kw
#15 **#8 OR #9 OR #10 OR #11 OR #12 OR #13 OR #14**

#16 MeSH descriptor: [Intensive Care Units] explode all trees
#17 (Care Unit, Intensive):ti,ab,kw
#18 (Care Units, Intensive):ti,ab,kw
#19 (Intensive Care Unit):ti,ab,kw
#20 (Unit, Intensive Care):ti,ab,kw
#21 (Units, Intensive Care):ti,ab,kw
#22 #16 **OR #17 OR #18 OR #19 OR #20 OR #21**
#23 #22 OR #15
#24 #23 AND #7
