# Supplementary material for: High Dose Vitamin D3 Supplementation Is Not Associated With Lower Mortality in Critically Ill Patients: A Meta-Analysis of Randomized Control Trials
Source: Front Nutr. 2022 May 4;9:762316. doi: 10.3389/fnut.2022.762316 (PMC9116294; doi:10.3389/fnut.2022.762316)
Supplement: Supplemental File 3 — The risk of bias of the included trials. [file Image_3.pdf]

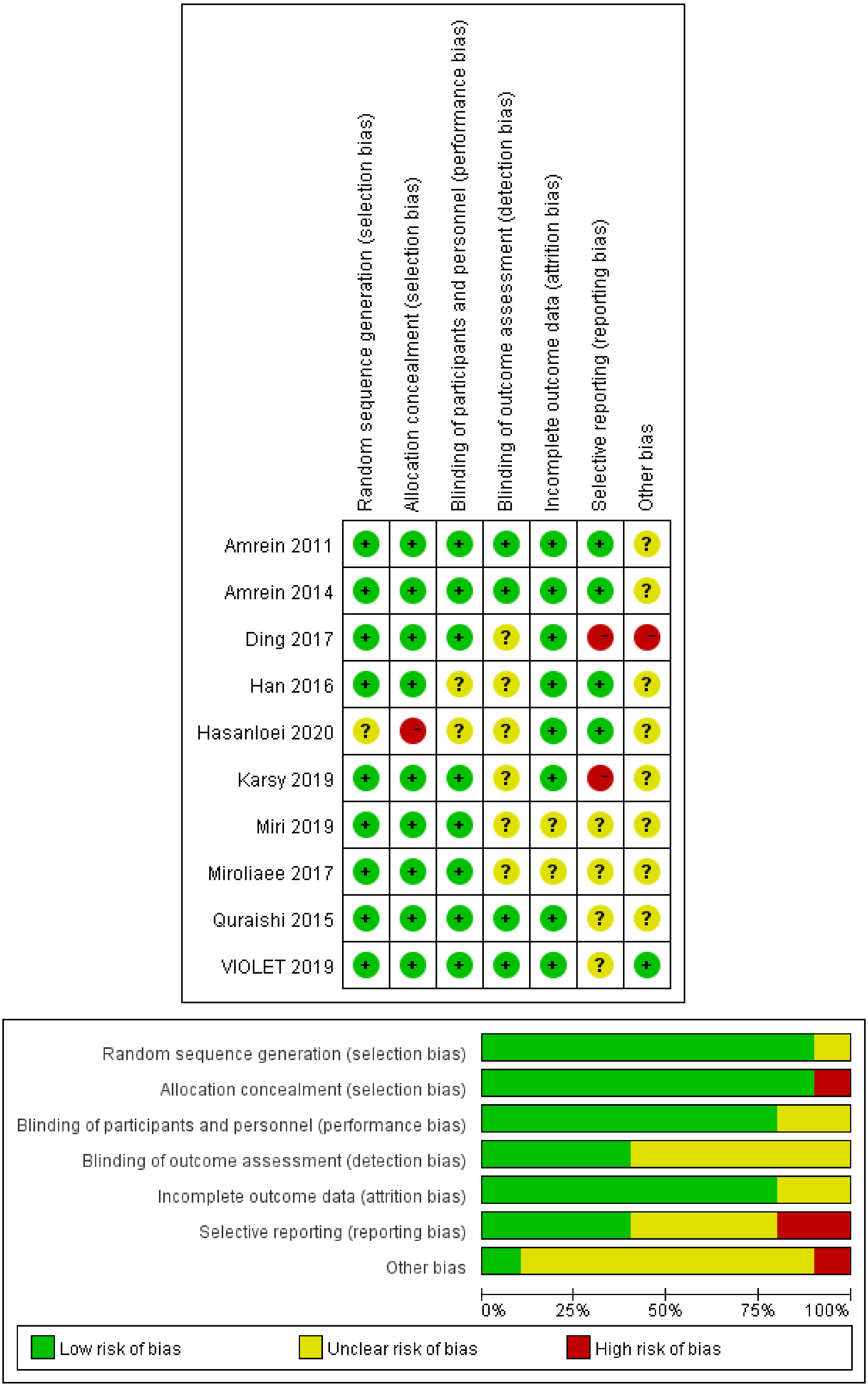
Random sequence generation (selection bias)
Allocation concealment (selection bias)
Blinding of participants and personnel (performance bias)
Blinding of outcome assessment (detection bias)
Incomplete outcome data (attrition bias)
Selective reporting (reporting bias)
Other bias
Amrein 2011
Amrein 2014
Ding 2017
Han 2016
Hasanloei 2020
Karsy 2019
Miri 2019
Miroliaee 2017
Quraishi 2015
VIOLET 2019
0%
25%
50%
75%
100%
Low risk of bias
Unclear risk of bias
High risk of bias

Supplemental files 3**.** The risk of bias of included trails
