# Supplementary material for: High Dose Vitamin D3 Supplementation Is Not Associated With Lower Mortality in Critically Ill Patients: A Meta-Analysis of Randomized Control Trials
Source: Front Nutr. 2022 May 4;9:762316. doi: 10.3389/fnut.2022.762316 (PMC9116294; doi:10.3389/fnut.2022.762316)
Supplement: Supplemental File 4 — Risk of bias assessment of the included trials. [file Image_4.pdf]

Risk of bias assessment of the included trials

1. Amrein 2011

Bias	Authors' judgement	Support for judgement
**Random sequence generation (selection bias)**	**LOW**	**Quote** "The study medication was prepared, labelled and randomized by a pharmacist and physician not involved in the trial. **The randomization was performed with sealed envelopes a**nd all aspects of the trial were performed in a double-blind fashion" (page2)
**Allocation concealment (selection bias)**	**LOW**	**Quote** "The study medication was prepared, labelled and randomized by a pharmacist and physician not involved in the trial. **The randomization was performed with sealed envelopes a**nd all aspects of the trial were performed in a double-blind fashion" (page2)
**Blinding of participants and personnel (performance bias)**	**LOW**	**Quote** "The study medication was prepared, labelled and randomized by a pharmacist and physician not involved in the trial. The randomization was performed with sealed envelopes and **all aspects of the trial were performed in a double-blind fashion"** (page2)
**Blinding of outcome assessment (detection bias)**	**LOW**	**Quote** "The study medication was prepared, labelled and randomized by a pharmacist and physician not involved in the trial. The randomization was performed with sealed envelopes and **all aspects of the trial were performed in a double-blind fashion"** (page2)
**Incomplete outcome data (attrition bias)**	**LOW**	**Comment** All the data was reported
**Selective reporting (reporting bias)**	**LOW**	**Comment** All the outcomes were reported
**Other bias**	**UNCLEAR**	**Comment** sample size was relatively small

2. Amrein 2014

Bias	Authors' judgement	Support for judgement
**Random sequence generation (selection bias)**	**LOW**	**Quote** *"Patientswere randomly assigned to either a placebo group or vitamin D3 group in a 1:1 ratio (Figure 1),* ***using the Randomizer for Clinical Trials tool developed at the Medical University of Graz****"* (page1521)
**Allocation concealment (selection bias)**	**LOW**	**Quote** *"Patientswere randomly assigned to either a placebo group or vitamin D3 group in a 1:1 ratio (Figure 1),* ***using the Randomizer for Clinical Trials tool developed at the Medical University of Graz****"* (page1521)
**Blinding of participants and personnel (performance bias)**	**LOW**	**Quite** *"All trial participants, investigators, and assessors were unaware of the assigned intervention."* (page1521)
**Blinding of outcome assessment (detection bias)**	**LOW**	**Comment** *Investigators who were blinded to study group assignments collected data.* (page1522)
**Incomplete outcome data (attrition bias)**	**LOW**	**Comment** No outcome data was missing.
**Selective reporting (reporting bias)**	**LOW**	**Comment** All the outcomes were reported.
**Other bias**	**UNCLEAR**	**Comment** Unclear

3. Ding 2017

Bias	Authors' judgement	Support for judgement
**Random sequence generation (selection bias)**	**LOW**	**Quote** *"……按随机数字表法分为 D3 治疗组和安慰剂组……" (page107)* *"The patients were randomly divided into the D3 treatment group and the placebo group by the random number table method "*
**Allocation concealment (selection bias)**	**LOW**	**Quote** *"…所有受试者进行随机分配隐藏…"(page107)* **"**All subjects were randomly assigned to hide**"**
**Blinding of participants and**	**LOW**	**Quote "…双盲…"***(page107)*

**personnel (performance bias)**		"…double blind…"
**Blinding of outcome assessment (detection bias)**	**UNCLEAR**	**Comment** There is insufficient information to judge the risk level
**Incomplete outcome data (attrition bias)**	**LOW**	**Comment** There was no missing data in the outcomes
**Selective reporting (reporting bias)**	**High**	**Comment** 28 day mortality was not the primary outcome
**Other bias**	**High**	**Comment** "…另外选择同期20 例全身炎症反应综合征（SIRS）患者和20 例健康体检志愿者作为对照…" "Meanwhile, 20 patients with systemic inflammatory response syndrome (SIRS) and 20 healthy volunteers were selected as the control group"

4. Han 2016

Bias	Authors' judgement	Support for judgement
**Random sequence generation (selection bias)**	**LOW**	**Quote** "Treatment groups were assigned by a blinded block randomization schedule overseen by biostatisticians of the Atlanta Clinical and Translational Science Institute (ACTSI) biostatistics core." (page 60)
**Allocation concealment (selection bias)**	**LOW**	**Comment** "Treatment groups were assigned by a blinded block randomization schedule overseen by biostatisticians of the Atlanta Clinical and Translational Science Institute (ACTSI) biostatistics core." (page 60)
**Blinding of participants and personnel (performance bias)**	**LOW**	**Comment** "Treatment groups were assigned by a blinded block randomization schedule overseen by biostatisticians of the Atlanta Clinical and Translational Science Institute (ACTSI) biostatistics core." "With the exception of the pharmacists, all study staff were blinded to the group allocation."(page 60)
**Blinding of outcome assessment**	**UNCLEAR**	**Comment** The blinding of outcomes was not mentioned in the main text.

**(detection bias)**		
**Incomplete outcome data (attrition bias)**	**LOW**	**Comment** All patient data reported
**Selective reporting (reporting bias)**	**LOW**	**Comment** All outcomes reported
**Other bias**	**UNCLEAR**	**Comment** Sample size relatively small

5. Hasanloei 2020

Bias	Authors' judgement	Support for judgement
**Random sequence generation (selection bias)**	**UNCLEAR**	**Quote** The study was registered in Iranina registry of clinical trials, but we can not get the detail information
**Allocation concealment (selection bias)**	**HIGH**	**Quote** the design of the study was not double-blinded
**Blinding of participants and personnel (performance bias)**	**UNCLEAR**	**Quote T**here is insufficient information to judge the risk level
**Blinding of outcome assessment (detection bias)**	**UNCLEAR**	**Comment** There is insufficient information to judge the risk level
**Incomplete outcome data (attrition bias)**	**LOW**	**Comment** There was no missing data in the outcomes
**Selective reporting (reporting bias)**	**High**	**Comment** The study was registered in Iranina registry of clinical trials
**Other bias**	**UNCLEAR**	**Comment** There is insufficient information to judge the risk level

6. Karsy 2019

Bias	Authors' judgement	Support for judgement
**Random sequence generation (selection bias)**	**LOW**	**Quote** “patients were randomized in a 1:1 manner using a random number generator.”
**Allocation concealment (selection bias)**	**LOW**	**Quote** “patients were randomized in a 1:1 manner using a random number generator, which was managed by the investigational drug pharmacy.”
**Blinding of participants and personnel (performance bias)**	**LOW**	**Quote** “blinded to the patients and investigators”
**Blinding of outcome assessment (detection bias)**	**UNCLEAR**	**Quote** there is insufficient information to judge the risk level
**Incomplete outcome data (attrition bias)**	**LOW**	**Comment** There was no missing data in the outcomes
**Selective reporting (reporting bias)**	**HIHG**	**Comment** 28 day mortality was not the primary outcome
**Other bias**	**UNCLEAR**	**Comment t**here is insufficient information to judge the risk level

7. Quraishi 2015		
**Bias**	**Authors' judgement**	**Support for judgement**
**Random sequence generation (selection bias)**	**LOW**	**Quote** *The Research Pharmacy was responsible for establishing the study code using a computer-generated block randomization algorithm, preparing syringes containing the study drug (page 1930)*
**Allocation concealment (selection bias)**	**LOW**	**Quote** *The Research Pharmacy was responsible for establishing the study code using a computer-generated block randomization algorithm, preparing syringes containing the study drug (page 1930)*
**Blinding of participants and personnel (performance bias)**	**LOW**	**Quote** *The Research Pharmacy was responsible for establishing the study code using a computer-generated block randomization algorithm, preparing syringes containing the study drug (page 1930)*
**Blinding of outcome assessment (detection bias)**	**LOW**	**Quote** *"The 30-day mortality rate for the analyticcohort was confirmed by reviewing individual medical recordsand cross-referencing each case using the Social Security Death Index Master File up to 90 days following hospital discharge" (page 1930)*
**Incomplete outcome data (attrition bias)**	**LOW**	**Comment** *The 30-day mortality rate for the analytic cohort was confirmed by reviewing individual medical records and cross-referencing each case using the Social Security Death Index Master File up to 90 days following hospital discharge (page 1930)*
**Selective reporting (reporting bias)**	**UNCLEAR**	**Comment** *Insufficient information*
**Other bias**	**UNCLEAR**	**Comment** None.

8. Miroliaee 2017		
**Bias**	**Authors' judgement**	**Support for judgement**
**Random sequence generation (selection bias)**	**LOW**	**Quote** “The "RAND" command of the Microsoft Excel® 2013, was used for randomized allocation of the patients” page 1256
**Allocation concealment (selection bias)**	**LOW**	**Quote** “The "RAND" command of the Microsoft Excel® 2013, was used for randomized allocation of the patients” page 1256
**Blinding of participants and personnel (performance bias)**	**LOW**	**Comment** double blind
**Blinding of outcome assessment (detection bias)**	**UNCLEAR**	**Comment** There is insufficient information to judge the risk level
**Incomplete outcome data (attrition bias)**	**UNCLEAR**	**Comment** There is insufficient information to judge the risk level
**Selective reporting (reporting bias)**	**UNCLEAR**	**Comment** There is insufficient information to judge the risk level
**Other bias**	**UNCLEAR**	**Comment** Sample size relatively small

9. Miri 2019		
**Bias**	**Authors' judgement**	**Support for judgement**
**Random sequence generation (selection bias)**	**LOW**	**Quote** *"…in 1:1 ratio by permuted block randomization." page1068*
**Allocation concealment (selection bias)**	**LOW**	**Quote** *"…in 1:1 ratio by permuted block randomization." page1068*
**Blinding of participants and personnel (performance bias)**	**LOW**	**Quote** *"double blind"*
**Blinding of outcome assessment (detection bias)**	**UNCLEAR**	**Quote** Not mentioned
**Incomplete outcome data (attrition bias)**	**UNCLEAR**	**Comment** Not mentioned
**Selective reporting (reporting bias)**	**UNCLEAR**	**Comment** Not mentioned
**Other bias**	**UNCLEAR**	**Comment** None.

10. VIOLET 2019		
**Bias**	**Authors' judgement**	**Support for judgement**
**Random sequence generation (selection bias)**	**LOW**	**Quote** *"We used a central electronic system and permuted blocks to randomly assign eligible patients in a 1:1 ratio, stratified according to site."*
**Allocation concealment (selection bias)**	**LOW**	**Quote** *"We used a central electronic system and permuted blocks to randomly assign eligible patients in a 1:1 ratio, stratified according to site."*
**Blinding of participants and personnel (performance bias)**	**LOW**	**Quote** *"double blind"*
**Blinding of outcome assessment (detection bias)**	**LOW**	**Quote** *Outcomes are unlikely to be affected by the absence of blindness*

**Incomplete outcome data (attrition bias)**	**LOW**	**Comment** All groups had no data lost.
**Selective reporting (reporting bias)**	**UNCLEAR**	**Comment** Not mentioned
**Other bias**	**LOW**	**Comment** None.
