# Supplementary material for: High Dose Vitamin D3 Supplementation Is Not Associated With Lower Mortality in Critically Ill Patients: A Meta-Analysis of Randomized Control Trials
Source: Front Nutr. 2022 May 4;9:762316. doi: 10.3389/fnut.2022.762316 (PMC9116294; doi:10.3389/fnut.2022.762316)
Supplement: Supplemental File 5 — Publication bias of mortality truncated to day 28 and day 90. [file Image_5.pdf]

A Publication bias of mortality truncated to day 28

Egger's test

Std_Eff	Coef.	Std. Err.	t	P>|t|	[95% Conf. Interval]	
**slope**	**.1851247**	**.1806771**	**1.02**	**0.336**	**-.2315175**	**.601767**
**bias**	**-1.147516**	**.617051**	**-1.86**	**0.100**	**-2.570438**	**.2754066**

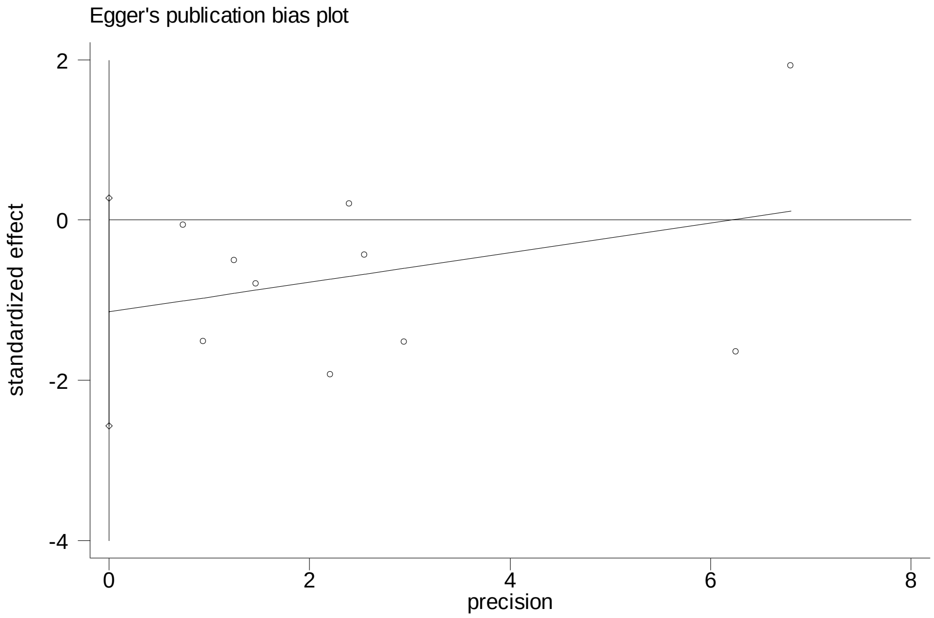

B Publication bias of mortality truncated to day 90

Egger's test

Std_Eff	Coef.	Std. Err.	t	P>|t|	[95% Conf. Interval]	
slope	.0630992	.1148528	0.55	0.598	-.2017517	.3279502
bias	-.924563	.4875689	-1.90	0.095	-2.048899	.199773

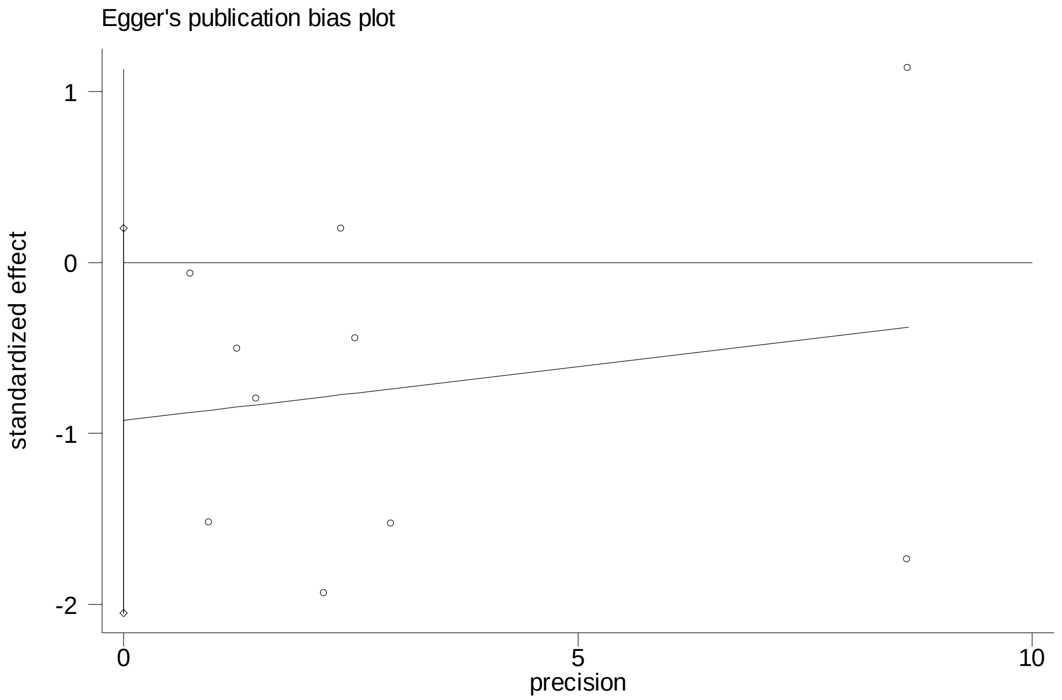
Egger's publication bias plot
standardized effect
1
0
-1
-2
0
5
10
precision
