# Supplementary material for: High Dose Vitamin D3 Supplementation Is Not Associated With Lower Mortality in Critically Ill Patients: A Meta-Analysis of Randomized Control Trials
Source: Front Nutr. 2022 May 4;9:762316. doi: 10.3389/fnut.2022.762316 (PMC9116294; doi:10.3389/fnut.2022.762316)

Sensitivity analysis

Mortality (truncated to day-28) sensitive analysis

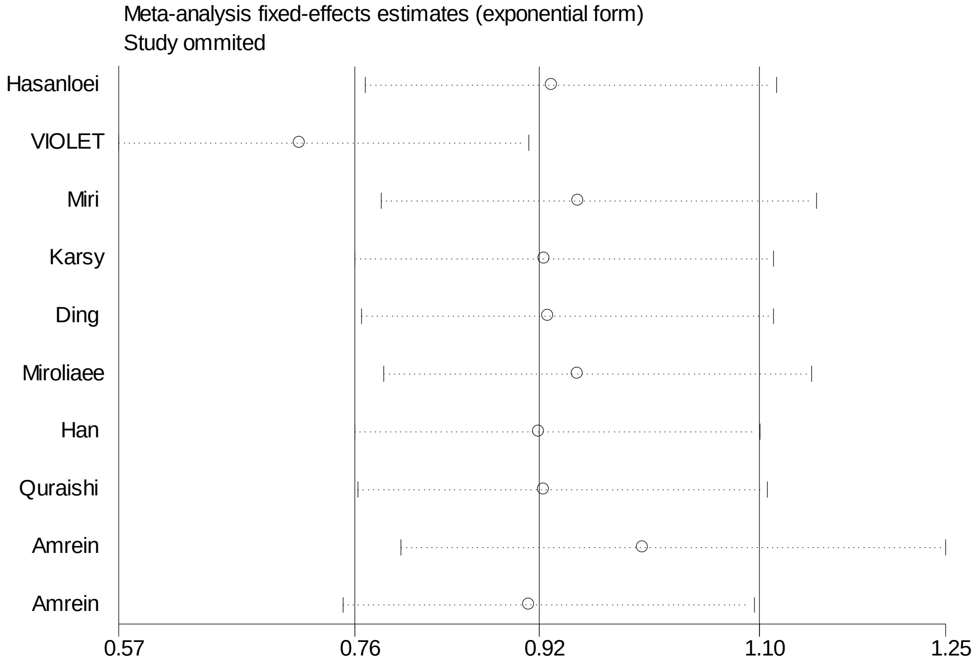

Mortality (truncated to day-90) sensitive analysis

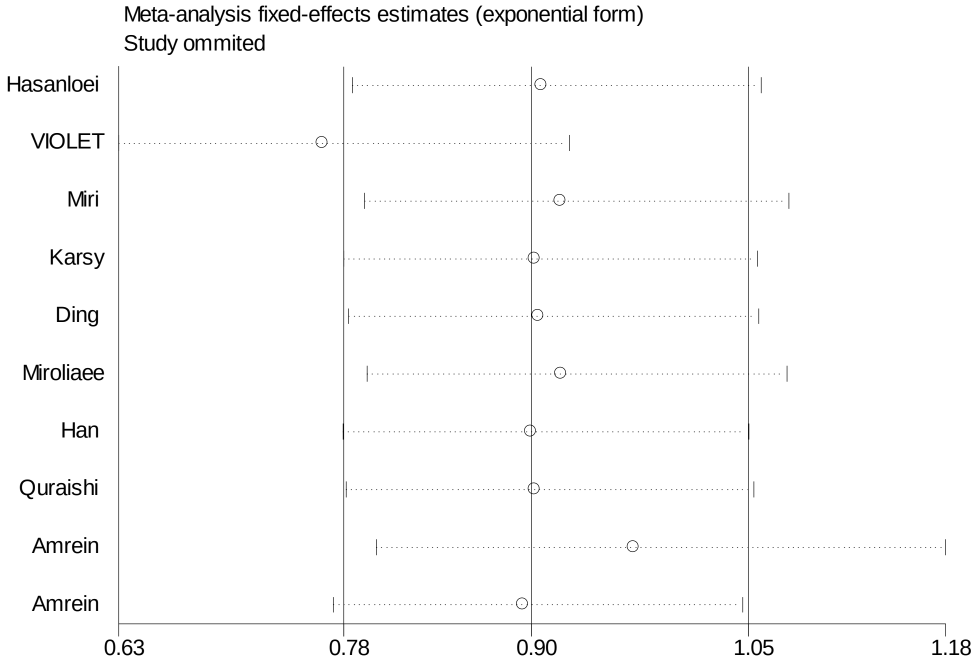

Supplement: Supplemental File 6 — Sensitivity analysis. [file Image_6.pdf]
