# Supplementary material for: High Dose Vitamin D3 Supplementation Is Not Associated With Lower Mortality in Critically Ill Patients: A Meta-Analysis of Randomized Control Trials
Source: Front Nutr. 2022 May 4;9:762316. doi: 10.3389/fnut.2022.762316 (PMC9116294; doi:10.3389/fnut.2022.762316)
Supplement: Supplemental File 7 — GRADE profile for assessing the quality of evidence for vitamin D3 in critically ill patients. [file Image_7.pdf]

**Prognosis for [Mortality]**

**Patient or population:** patients with [Mortality]
**Settings:**
**Intervention:** Prognosis

| Outcomes | Illustrative comparative risks* (95% CI) Assumed risk Control | Corresponding risk Prognosis | Relative effect (95% CI) | No of Participants (studies) | Quality of the evidence (GRADE) | Comments |
|---|---|---|---|---|---|---|
| **mortality - mortality 28 day** | **Study population** | | **RR 0.93** (0.78 to 1.11) | 2058 (10 studies) | ⊕⊕⊖⊖ **low**[1,2,3] | |
| | **188 per 1000** | **174 per 1000** (146 to 208) | | | | |
| | **Moderate** | | | | | |
| | **247 per 1000** | **230 per 1000** (193 to 274) | | | | |
| **mortality - mortality 90 day** | **Study population** | | **RR 0.91** (0.79 to 1.05) | 2058 (10 studies) | ⊕⊕⊖⊖ **low**[1,2,3] | |
| | **260 per 1000** | **236 per 1000** (205 to 273) | | | | |
| | **Moderate** | | | | | |
| | **254 per 1000** | **231 per 1000** (201 to 267) | | | | |

*The basis for the **assumed risk** (e.g. the median control group risk across studies) is provided in footnotes. The **corresponding risk** (and its 95% confidence interval) is based on the assumed risk in the comparison group and the **relative effect** of the intervention (and its 95% CI).

**CI:** Confidence interval; **RR:** Risk ratio;

GRADE Working Group grades of evidence
**High quality:** Further research is very unlikely to change our confidence in the estimate of effect.
**Moderate quality:** Further research is likely to have an important impact on our confidence in the estimate of effect and may change the estimate.
**Low quality:** Further research is very likely to have an important impact on our confidence in the estimate of effect and is likely to change the estimate.
**Very low quality:** We are very uncertain about the estimate.

[1] different vitamin D3 dosage

[2] I2=41% P=0.09 heterogeneity for mortality 28 day, I2=26% P=0.21 for mortality day 90

[3] mortality was not the primary outcome in some trails
