# Supplementary material for: High Dose Vitamin D3 Supplementation Is Not Associated With Lower Mortality in Critically Ill Patients: A Meta-Analysis of Randomized Control Trials
Source: Front Nutr. 2022 May 4;9:762316. doi: 10.3389/fnut.2022.762316 (PMC9116294; doi:10.3389/fnut.2022.762316)
Supplement: Supplemental File 8 — Trial sequential analysis revealing the optimal sample size for detecting the plausible effect of vitamin D3 use on mortality truncated to 28 days. [file Image_8.pdf]

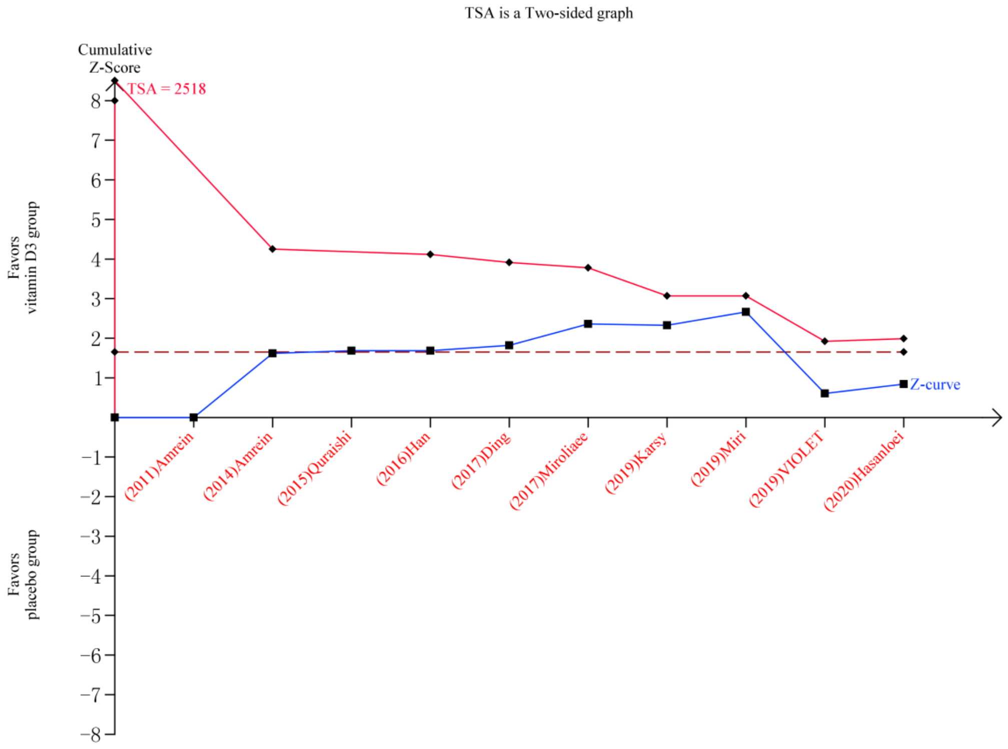

Supplemental files 8. Trial sequential analysis revealing the optimal sample size for detecting the plausible effect of vitamin D3 use in mortality truncated to 28 day.
