# Supplementary material for: High Dose Vitamin D3 Supplementation Is Not Associated With Lower Mortality in Critically Ill Patients: A Meta-Analysis of Randomized Control Trials
Source: Front Nutr. 2022 May 4;9:762316. doi: 10.3389/fnut.2022.762316 (PMC9116294; doi:10.3389/fnut.2022.762316)

Following factors were concluded in the univariate meta-regression analysis,

- Year when the study was published,
- Country of study conducted, USA, Iran, Austria, or Austrilia,
- No. of centers included in the study,
- No. of subjects included in the study,
- The dosage of vitamin D3 applied in the study
- The method of vitamin D3 administration, enteral or intramuscular,
-

Trial	Year	Country	No. of centers	Sample size, n	Vitamin D dosage (IU)	Method of administration
Hasanloei	2020	Iran	Single center	48	300,000	Intramuscular
VIOLET	2019	USA	Multicenter	1078	540,000	Enteral
Miri	2019	Iran	Single center	40	300,000	Intramuscular
Karsy	2019	USA	Single center	267	540,000	Enteral
Ding	2017	China	Single center	57	300,000	Intramuscular
Miroliaee	2017	Iran	Multicenter	46	300,000	Intramuscular
Han	2016	USA	Multicenter	30	500,000	Enteral
Quraishi	2015	USA	Single center,	30	400,000	Enteral
Amrein	2014	Austria	Single center	492	540,000	Enteral
Amrein	2011	Austria	Single center	25	540,000	Enteral

Year P=0.837

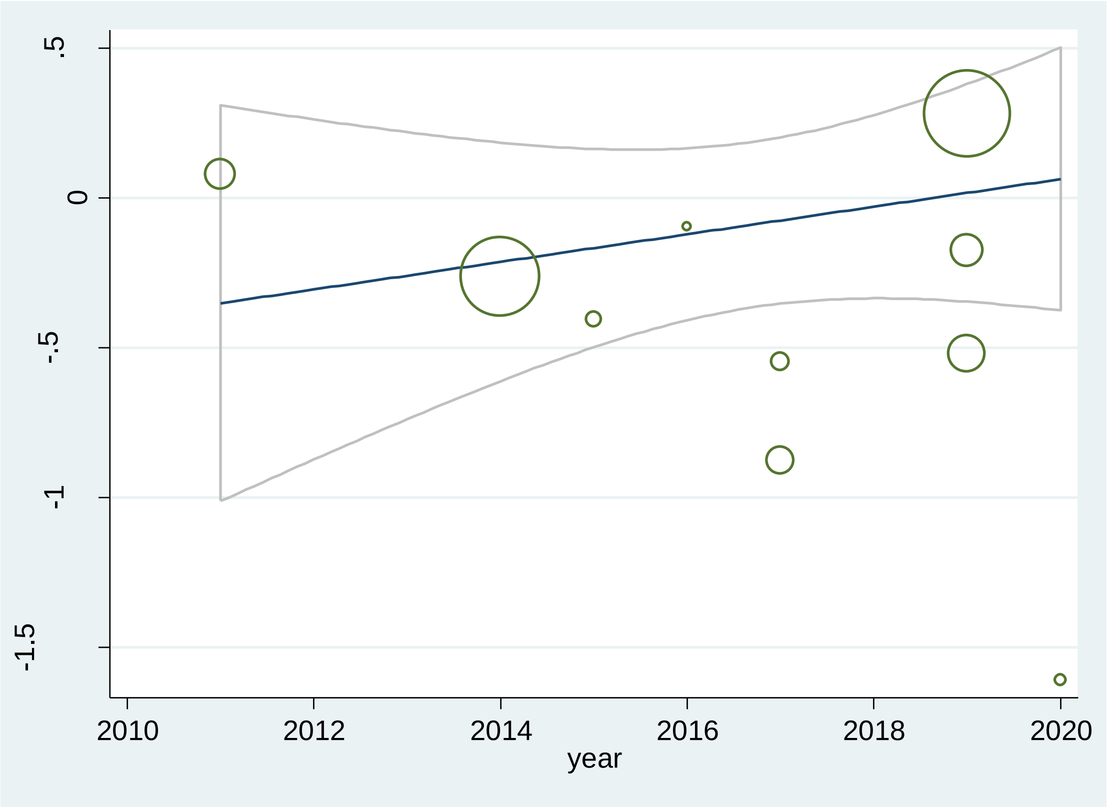

Country P=0.212 1=Iran 2=USA 3=CHINA 4=AUSTRIA

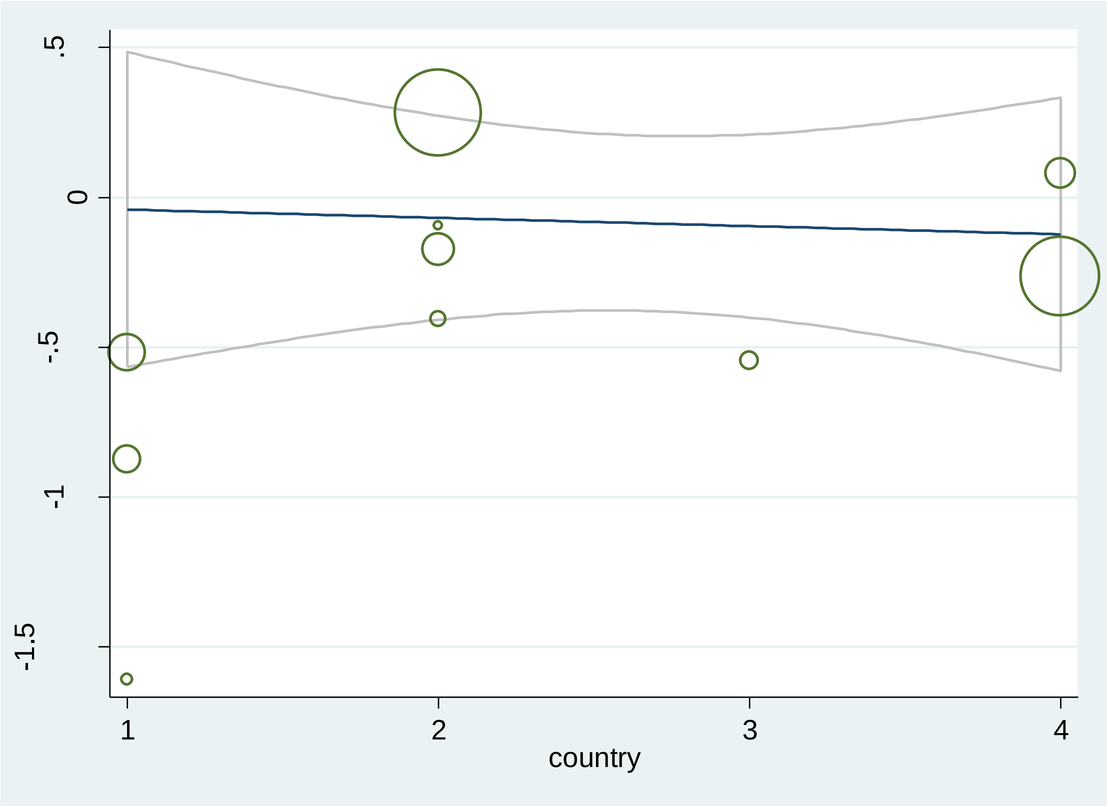

Number of centers p=0.717 1=single 2=multi

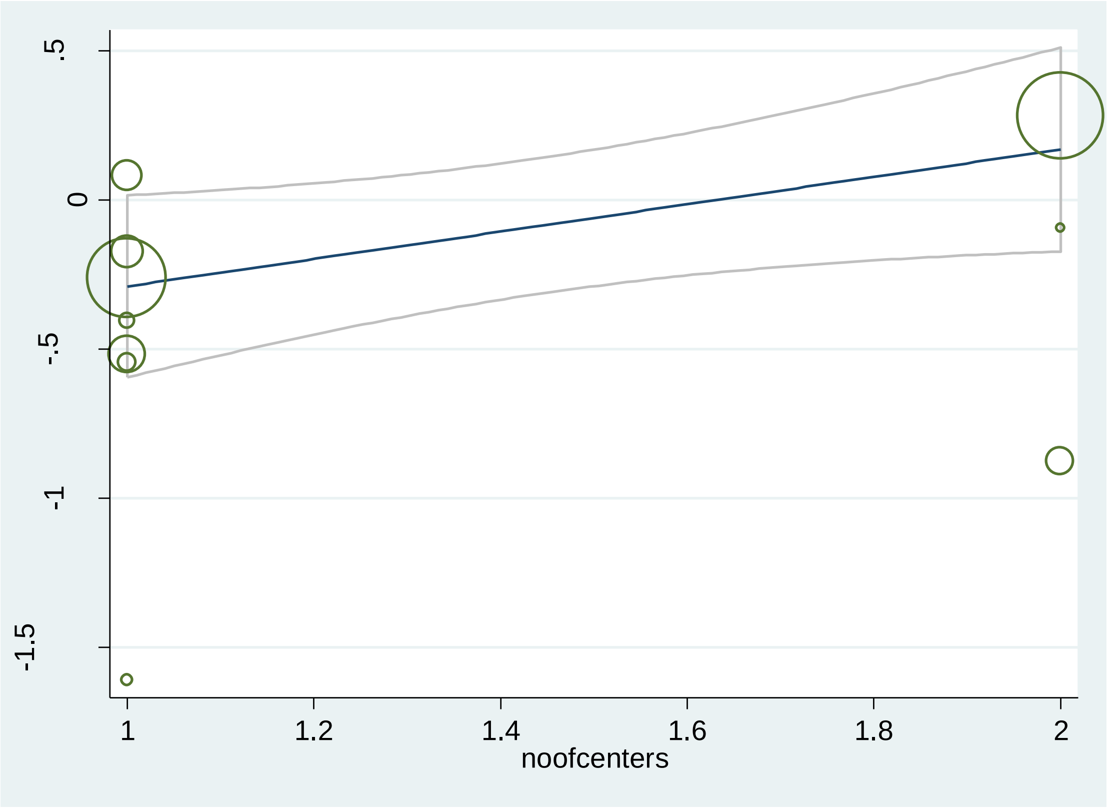

Vitamin D3 dosage p=0.039

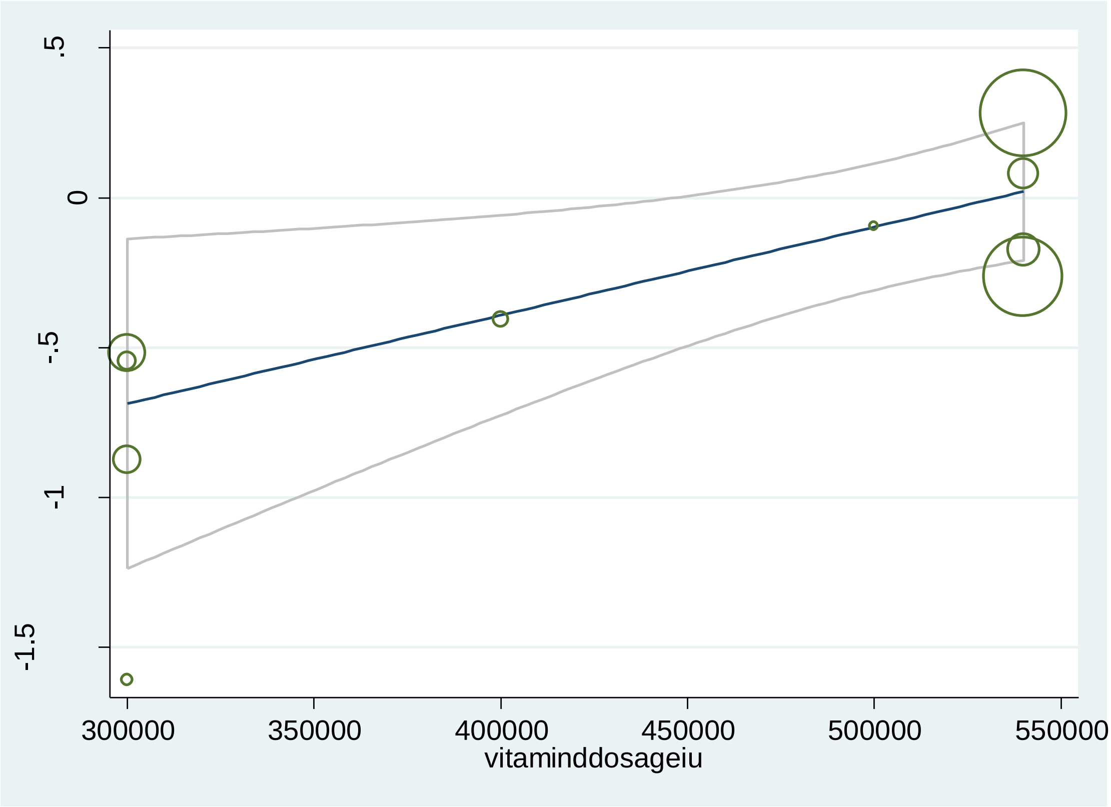

Sample size P=0.012

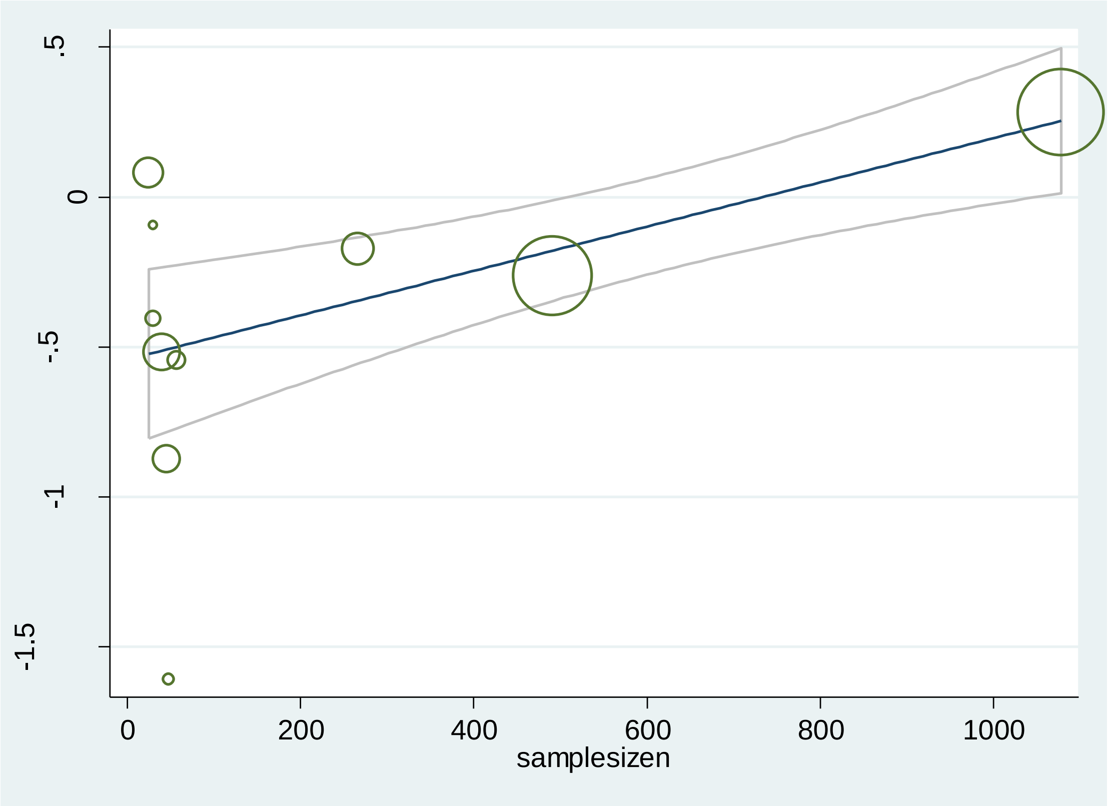

Method of Vd3 administration P=0.041

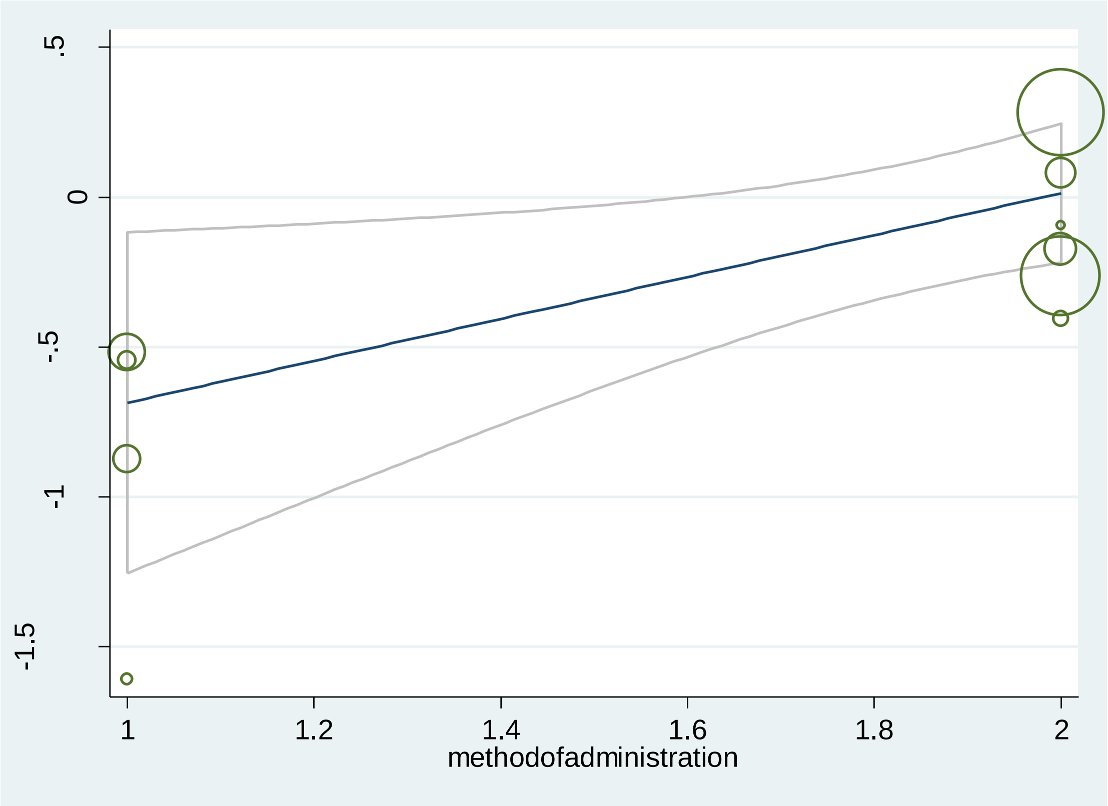

Supplement: Supplemental File 9 — Univariate meta-regression analysis. [file Image_9.pdf]
