# Supplementary material for: High Dose Vitamin D3 Supplementation Is Not Associated With Lower Mortality in Critically Ill Patients: A Meta-Analysis of Randomized Control Trials
Source: Front Nutr. 2022 May 4;9:762316. doi: 10.3389/fnut.2022.762316 (PMC9116294; doi:10.3389/fnut.2022.762316)
Supplement: Supplemental File 10 — Subgroup analysis. Patients were divided by dose of vitamin D3 (300,000 IU, 400,000 IU, and 540,000 IU). [file Image_10.pdf]

A

Study or Subgroup	Vitamin D3 Events	Vitamin D3 Total	Placebo Events	Placebo Total	Weight	Risk Ratio M-H, Fixed, 95% CI
**1.8.1 Vitamin D 300,000IU**						
Ding 2017	3	29	5	28	2.6%	0.58 [0.15, 2.20]
Hasanloei 2020	1	24	5	24	2.6%	0.20 [0.03, 1.59]
Miri 2019	8	22	11	18	6.3%	0.60 [0.31, 1.16]
Miroliaee 2017	5	24	11	22	5.9%	0.42 [0.17, 1.01]
**Subtotal (95% CI)**		**99**		**92**	**17.4%**	**0.47 [0.29, 0.77]**
Total events	17		32			
Heterogeneity: Chi² = 1.29, df = 3 (P = 0.73); I² = 0%						
Test for overall effect: Z = 2.98 (P = 0.003)						
**1.8.2 Vitamin D 400,000 IU**						
Quraishi 2015	2	10	3	10	1.5%	0.67 [0.14, 3.17]
**Subtotal (95% CI)**		**10**		**10**	**1.5%**	**0.67 [0.14, 3.17]**
Total events	2		3			
Heterogeneity: Not applicable						
Test for overall effect: Z = 0.51 (P = 0.61)						
**1.8.3 Vitamin D 540,000 IU**						
Amrein 2011	6	12	6	13	3.0%	1.08 [0.48, 2.45]
Amrein 2014	52	237	68	238	35.1%	0.77 [0.56, 1.05]
Han 2016	1	11	1	10	0.5%	0.91 [0.07, 12.69]
Karsy 2019	11	134	13	133	6.7%	0.84 [0.39, 1.81]
VIOLET 2019	92	531	69	528	35.7%	1.33 [0.99, 1.77]
**Subtotal (95% CI)**		**925**		**922**	**81.1%**	**1.03 [0.85, 1.26]**
Total events	162		157			
Heterogeneity: Chi² = 6.63, df = 4 (P = 0.16); I² = 40%						
Test for overall effect: Z = 0.32 (P = 0.75)						
**Total (95% CI)**		**1034**		**1024**	**100.0%**	**0.93 [0.78, 1.11]**
Total events	181		192			
Heterogeneity: Chi² = 15.14, df = 9 (P = 0.09); I² = 41%						
Test for overall effect: Z = 0.79 (P = 0.43)						
Test for subgroup differences: Chi² = 8.51, df = 2 (P = 0.01), I² = 76.5%						

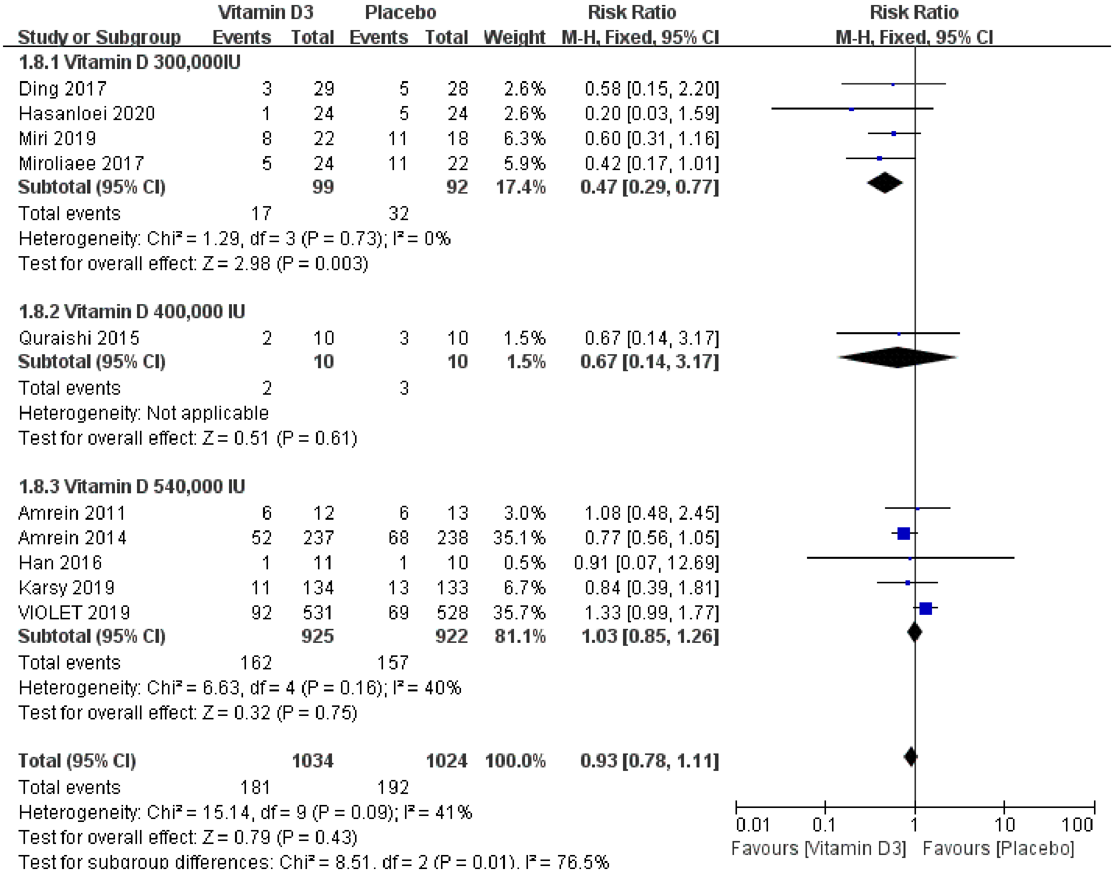

B

Study or Subgroup	Vitamin D3 Events	Vitamin D3 Total	Placebo Events	Placebo Total	Weight	Risk Ratio M-H, Fixed, 95% CI
**1.9.1 Vitamin D 300,000IU**						
Ding 2017	3	29	5	28	1.9%	0.58 [0.15, 2.20]
Hasanloei 2020	1	24	5	24	1.9%	0.20 [0.03, 1.59]
Miri 2019	8	22	11	18	4.5%	0.60 [0.31, 1.16]
Miroliaee 2017	5	24	11	22	4.3%	0.42 [0.17, 1.01]
**Subtotal (95% CI)**		**99**		**92**	**12.6%**	**0.47 [0.29, 0.77]**
Total events	17		32			
Heterogeneity: Chi² = 1.29, df = 3 (P = 0.73); I² = 0%						
Test for overall effect: Z = 2.98 (P = 0.003)						
**1.9.2 Vitamin D 400,000 IU**						
Quraishi 2015	4	11	2	10	0.8%	1.82 [0.42, 7.87]
**Subtotal (95% CI)**		**11**		**10**	**0.8%**	**1.82 [0.42, 7.87]**
Total events	4		2			
Heterogeneity: Not applicable						
Test for overall effect: Z = 0.80 (P = 0.42)						
**1.9.3 Vitamin D 540,000 IU**						
Amrein 2011	6	12	6	13	2.2%	1.08 [0.48, 2.45]
Amrein 2014	83	237	102	238	38.2%	0.82 [0.65, 1.03]
Han 2016	1	11	1	10	0.4%	0.91 [0.07, 12.69]
Karsy 2019	11	134	13	133	4.9%	0.84 [0.39, 1.81]
VIOLET 2019	125	531	109	528	41.0%	1.14 [0.91, 1.43]
**Subtotal (95% CI)**		**925**		**922**	**86.6%**	**0.98 [0.84, 1.14]**
Total events	226		231			
Heterogeneity: Chi² = 4.38, df = 4 (P = 0.36); I² = 9%						
Test for overall effect: Z = 0.28 (P = 0.78)						
**Total (95% CI)**		**1035**		**1024**	**100.0%**	**0.92 [0.80, 1.07]**
Total events	247		265			
Heterogeneity: Chi² = 12.81, df = 9 (P = 0.17); I² = 30%						
Test for overall effect: Z = 1.10 (P = 0.27)						
Test for subgroup differences: Chi² = 8.47, df = 2 (P = 0.01), I² = 76.4%						

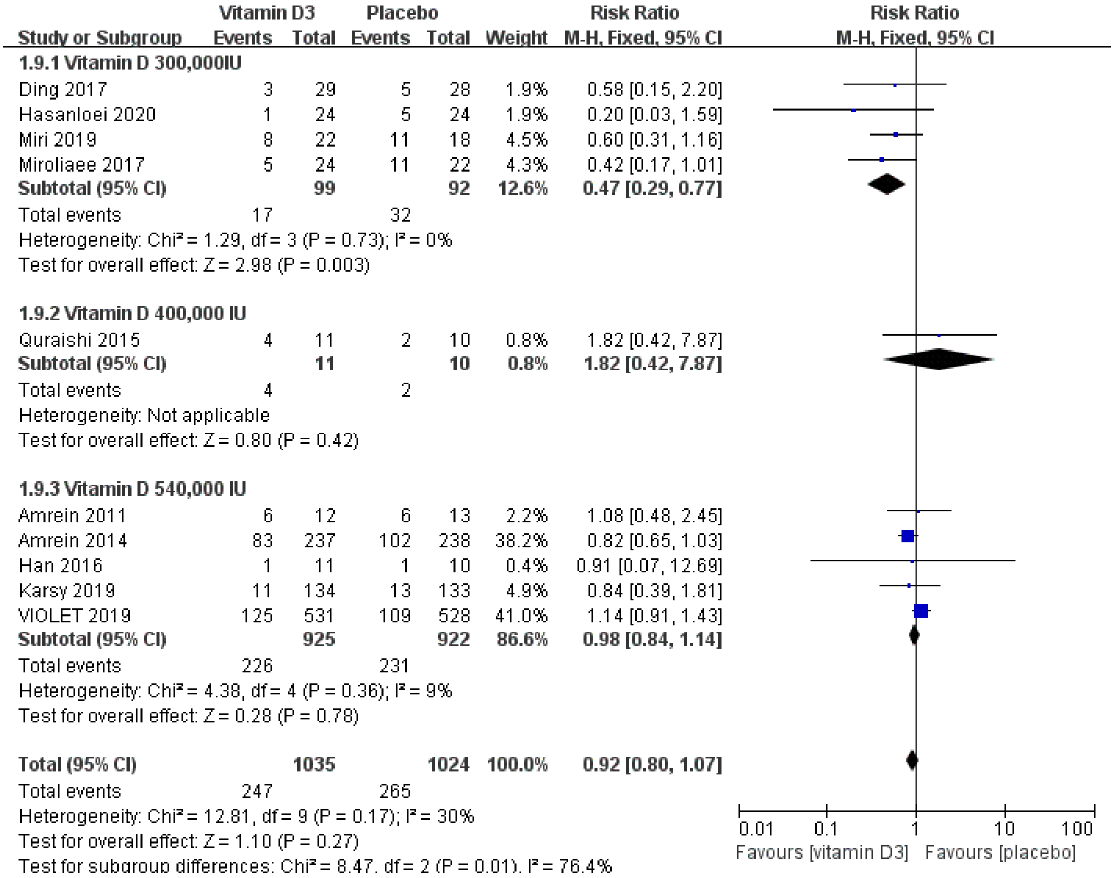

Supplemental files 10. The effect of vitamin D3 on mortality truncated to day 28 (**A**) and day 90 (**B**) in subgroup adult ICU patients according vitamin D3 dosage.
