# Supplementary material for: High Dose Vitamin D3 Supplementation Is Not Associated With Lower Mortality in Critically Ill Patients: A Meta-Analysis of Randomized Control Trials
Source: Front Nutr. 2022 May 4;9:762316. doi: 10.3389/fnut.2022.762316 (PMC9116294; doi:10.3389/fnut.2022.762316)
Supplement: Supplemental File 11 — Subgroup analysis. Patients were divided by vitamin D3 administration route (enteral and intramuscular). [file Image_11.pdf]

A

Study or Subgroup	vitamin D Events	vitamin D Total	placebo Events	placebo Total	Weight	Odds Ratio M-H, Fixed, 95% CI
**1.10.1 Enteral administration**						
Amrein 2011	6	12	6	13	1.9%	1.17 [0.24, 5.62]
Amrein 2014	52	237	68	238	34.3%	0.70 [0.46, 1.07]
Han 2016	1	11	1	10	0.6%	0.90 [0.05, 16.59]
Karsy 2019	11	134	13	133	7.8%	0.83 [0.36, 1.91]
Quraishi 2015	2	10	3	10	1.6%	0.58 [0.07, 4.56]
VIOLET 2019	92	531	69	528	37.0%	1.39 [0.99, 1.96]
**Subtotal (95% CI)**		**935**		**932**	**83.1%**	**1.03 [0.81, 1.32]**
Total events	164		160			
Heterogeneity: Chi² = 6.90, df = 5 (P = 0.23); I² = 28%						
Test for overall effect: Z = 0.25 (P = 0.80)						
**1.10.2 Intramuscular administration**						
Ding 2017	3	29	5	28	3.0%	0.53 [0.11, 2.47]
Hasanloei 2020	1	24	5	24	3.1%	0.17 [0.02, 1.54]
Miri 2019	8	22	11	18	5.0%	0.36 [0.10, 1.32]
Miroliaee 2017	5	24	11	22	5.9%	0.26 [0.07, 0.96]
**Subtotal (95% CI)**		**99**		**92**	**16.9%**	**0.32 [0.15, 0.67]**
Total events	17		32			
Heterogeneity: Chi² = 0.88, df = 3 (P = 0.83); I² = 0%						
Test for overall effect: Z = 3.04 (P = 0.002)						
**Total (95% CI)**		**1034**		**1024**	**100.0%**	**0.91 [0.73, 1.15]**
Total events	181		192			
Heterogeneity: Chi² = 16.12, df = 9 (P = 0.06); I² = 44%						
Test for overall effect: Z = 0.79 (P = 0.43)						
Test for subgroup differences: Chi² = 8.77, df = 1 (P = 0.003), I² = 88.6%						

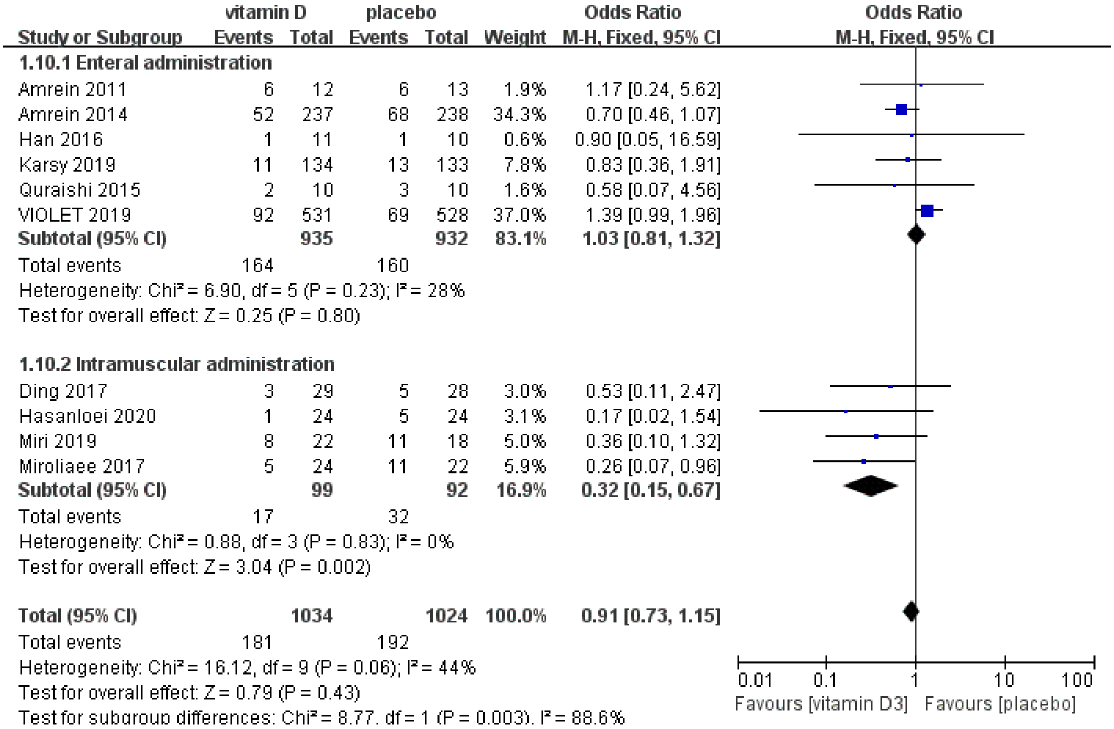

B

Study or Subgroup	vitamin D3 Events	vitamin D3 Total	placebo Events	placebo Total	Weight	Risk Ratio M-H, Fixed, 95% CI
**1.11.1 Enteral administration**						
Amrein 2011	6	12	6	13	2.2%	1.08 [0.48, 2.45]
Amrein 2014	83	237	102	238	38.2%	0.82 [0.65, 1.03]
Han 2016	1	11	1	10	0.4%	0.91 [0.07, 12.69]
Karsy 2019	11	134	13	133	4.9%	0.84 [0.39, 1.81]
Quraishi 2015	4	11	2	10	0.8%	1.82 [0.42, 7.87]
VIOLET 2019	125	531	109	528	41.0%	1.14 [0.91, 1.43]
**Subtotal (95% CI)**		**936**		**932**	**87.4%**	**0.99 [0.85, 1.15]**
Total events	230		233			
Heterogeneity: Chi² = 5.10, df = 5 (P = 0.40); I² = 2%						
Test for overall effect: Z = 0.18 (P = 0.86)						
**1.11.2 Intramuscular administration**						
Ding 2017	3	29	5	28	1.9%	0.58 [0.15, 2.20]
Hasanloei 2020	1	24	5	24	1.9%	0.20 [0.03, 1.59]
Miri 2019	8	22	11	18	4.5%	0.60 [0.31, 1.16]
Miroliaee 2017	5	24	11	22	4.3%	0.42 [0.17, 1.01]
**Subtotal (95% CI)**		**99**		**92**	**12.6%**	**0.47 [0.29, 0.77]**
Total events	17		32			
Heterogeneity: Chi² = 1.29, df = 3 (P = 0.73); I² = 0%						
Test for overall effect: Z = 2.98 (P = 0.003)						
**Total (95% CI)**		**1035**		**1024**	**100.0%**	**0.92 [0.80, 1.07]**
Total events	247		265			
Heterogeneity: Chi² = 12.81, df = 9 (P = 0.17); I² = 30%						
Test for overall effect: Z = 1.10 (P = 0.27)						
Test for subgroup differences: Chi² = 7.81, df = 1 (P = 0.005), I² = 87.2%						

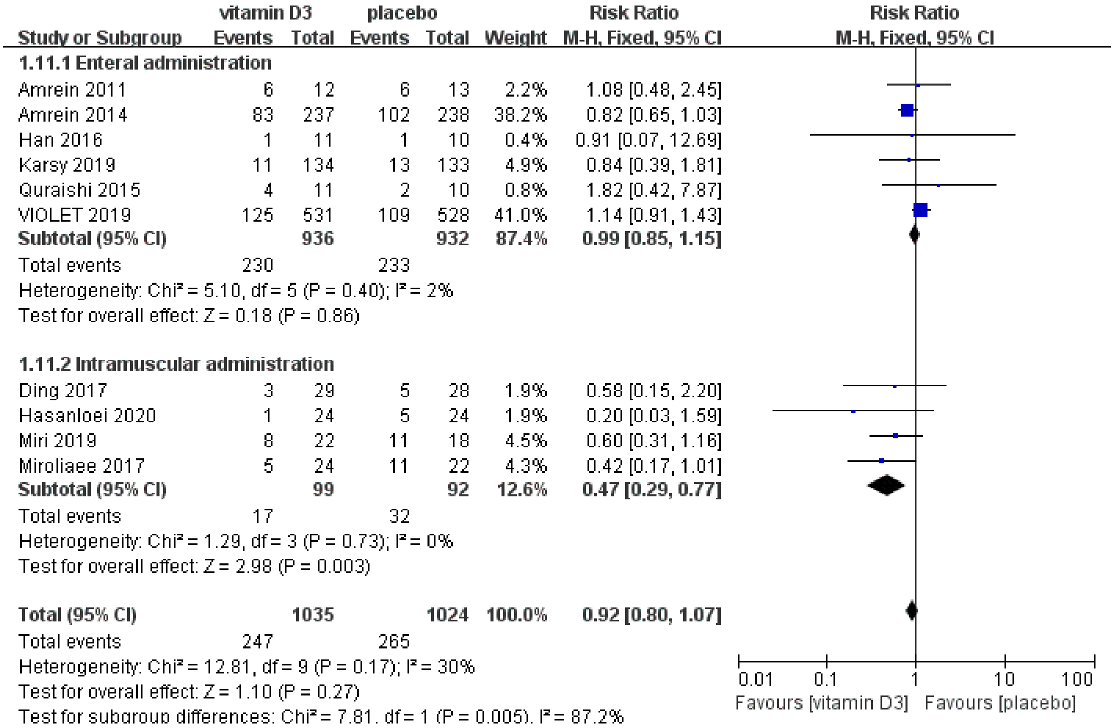

Supplemental files 11. The effect of vitamin D3 on mortality truncated to day 28 (**A**) and day 90 (**B**) in subgroup adult ICU patients according vitamin D3 administration route.
